# Perioperative Hemoglobin Decline After Deep Endometriosis Surgery: Association with Surgery Duration and Exploratory Analyses of Operative Extent

**DOI:** 10.3390/jcm15208084

**Published:** 2026-10-18

**Authors:** Krzysztof Nowak, Maja Mrugała, Weronika Ogonowska

**Affiliations:** 1 Clinical Department of Gynecologic Oncology, Prof. Tadeusz Koszarowski Opole Cancer Centre, 66A Katowicka Street, 45-061 Opole, Poland; maja.mrugala@gmail.com; 2 Clinical Department of Gynecologic Oncology, Institute of Medical Sciences, University of Opole, 48 Oleska Street, 45-052 Opole, Poland; 3 J. Gromkowski Voivodship Specialist Hospital in Wroclaw, 5 Koszarowa Street, 51-149 Wroclaw, Poland; wogonowska98@wp.pl

**Keywords:** deep endometriosis, hemoglobin, patient blood management, surgery duration, bowel resection, perioperative anemia

## Abstract

**Background/Objectives:** Deep endometriosis surgery may require bowel and multiorgan procedures, but reported blood loss and complications do not fully characterize postoperative hemoglobin decline, a relevant consideration for patient blood management. We aimed to quantify the frequency of a hemoglobin decline of ≥3 g/dL and its association with surgery duration, with secondary exploratory analyses of operative extent. **Methods:** This retrospective single-center study in Opole, Poland, linked registry and laboratory records for first eligible surgeries during 2020–2025. Decline was the last preoperative hemoglobin within 7 days minus the recorded nadir through postoperative day 3. The ≥3 g/dL threshold was operational, not a validated minimum clinically important difference. Multivariable models examined binary and continuous decline; exploratory analyses assessed operative extent. **Results:** Among 424 patients, preoperative hemoglobin was <12 g/dL in 41/419 (9.8%) and <13 g/dL in 152/419 (36.3%). Of 416 with both measurements, 156 (37.5%) met the endpoint, whose ascertainment depended on available recorded postoperative measurements. Each additional hour was associated with higher odds (odds ratio [OR], 1.61; 95% confidence interval [CI], 1.35–1.91), including without preoperative hemoglobin (OR, 1.50; 95% CI, 1.28–1.76). Continuous decline was 0.20 g/dL greater per hour (95% CI, 0.13–0.26). Among 379 patients with resection-multiplicity data, unadjusted endpoint frequencies were 14.5%, 38.3%, and 54.9% after no, one, and multiple full-thickness resections. **Conclusions:** Longer duration was associated with greater decline. Exploratory operative-extent gradients require prospective evaluation with standardized sampling before informing monitoring. Preoperative anemia remains relevant because the same decline produces a lower postoperative concentration.

## 1. Introduction

Surgery for deep endometriosis may combine rectosigmoid and upper-bowel procedures in a single session. For the operating surgeon, the practical question is which surgeries are most likely to be followed by a hemoglobin decline of at least 3 g/dL. This information may inform preoperative preparation and the timing of postoperative testing [1,2,3].

Preoperative anemia and perioperative hemoglobin decline represent different clinical problems. A lower preoperative concentration leaves less reserve after any given decline, so anemia should be investigated and treated when indicated. Surgical features may identify a different group: patients whose surgery is likely to be followed by a larger decline and who may benefit from planned postoperative monitoring. In an American College of Surgeons National Surgical Quality Improvement Program (ACS NSQIP) cohort of 12,836 women undergoing uterine surgery, preoperative anemia, defined as hematocrit < 36%, was present in 23.9% [4]. This provides gynecological context, although the procedure mix and hematocrit-based definition differ from those in deep endometriosis surgery.

The extent of surgery varies widely among patients. Guidelines recommend specialist-center treatment and an individualized multidisciplinary plan [5,6,7]. Previous studies have linked bowel resection and extensive disease with longer operating times, greater measured blood loss, and postoperative morbidity [8,9,10]. However, aggregate blood-loss or complication estimates cannot establish the proportion of patients with a defined ≥3 g/dL decline from baseline to the postoperative nadir. A directly comparable frequency cannot therefore be inferred from those endpoints.

Patient blood management (PBM) begins before surgery and continues through postoperative care. It combines identification and treatment of anemia, measures that limit avoidable blood loss, and appropriately timed blood counts [1,2]. However, transfusion is a treatment-dependent endpoint influenced by clinical thresholds, patient status, and local practice [11]. Many gynecologic prediction studies have nevertheless used transfusion as their endpoint [12,13], and published models vary substantially [14].

Our primary aim was to quantify the frequency of a hemoglobin decline of at least 3 g/dL and examine its association with surgery duration. Secondary objectives were to analyze decline as a continuous outcome, compare clinically recognizable categories of surgical type and extent, and test whether the duration finding persisted after preoperative hemoglobin was removed from the model.

This analysis extends previously published institutional surgical cohorts; at least 402 of the 424 participants appeared in one or more earlier series. The distinct questions and shared participants are mapped in Table S1.

## 2. Materials and Methods

### 2.1. Study Design and Setting

We conducted a single-center retrospective cohort study of patients who underwent surgery for deep endometriosis at the Clinical Department of Gynecologic Oncology, Prof. Tadeusz Koszarowski Opole Cancer Center, an academic affiliate of the Institute of Medical Sciences, University of Opole. Patients were usually admitted two days before surgery. Discharge timing was individualized according to the patient’s clinical condition, laboratory findings including C-reactive protein and procalcitonin, passage of stool, and body temperature. Data were drawn from the institutional deep-endometriosis registry, linked locally to laboratory records. The study period covered 1 January 2020 through 31 December 2025. Reporting followed the STROBE statement and the RECORD extension for routinely collected health data [15,16]. Completed reporting checklists accompany the revised manuscript.

### 2.2. Participants

Surgeries recorded in the institutional deep-endometriosis registry during the study period were eligible if they had a same-day link to the operative record, a recorded start time and duration, and organ-specific procedure information. Availability of hemoglobin measurements was not an eligibility criterion. When a patient had more than one eligible surgery, the earliest was selected as the index surgery. Each patient therefore contributed one surgery to the analysis.

This study included all eligible index operations within the specified period; no a priori sample-size calculation was performed. The selection flow begins with eligible linked operations; counts of registry records failing the earlier linkage or source-completeness criteria were not reconstructed.

### 2.3. Hemoglobin Measurements and Outcomes

The departmental routine was to obtain complete blood counts daily starting on postoperative day 1. The retrospective analysis used the recorded measurements available within the predefined windows; adherence to the daily sampling routine was not evaluated.

Preoperative hemoglobin (Hb) was the last measurement obtained from 7 days before surgery until the recorded start of surgery. The recorded postoperative nadir was the lowest available value after the end of surgery through postoperative day 3. All recorded Hb measurements within this window were eligible; selection was not restricted to one measurement per postoperative day. Postoperative day 0 was the operation calendar date. If the same minimum occurred more than once, the earliest sample determined the nadir day. No intraoperative hemoglobin measurements were available. Preoperative anemia was described at Hb < 12 g/dL; the additional <13 g/dL threshold was reported after peer review to align with International Consensus Conference on Anemia Management in Surgical Patients (ICCAMS) surgical guidance [2]. Both used the same preoperative measurement and denominator.

The primary outcome was an absolute hemoglobin decline of at least 3 g/dL, calculated as preoperative hemoglobin minus the postoperative nadir. This operational threshold was selected before the feasibility analysis but is not a validated minimum clinically important difference. Continuous decline was a key secondary outcome. Other secondary outcomes were a decline of at least 4 g/dL, a postoperative nadir below 9 g/dL and below 8 g/dL, and red blood cell (RBC) transfusion during the index hospitalization.

Recorded red blood cell transfusions were extracted from the hospital medical records. Details of transfusion ascertainment are provided in the Supplementary Methods.

### 2.4. Surgical Variables and Other Covariates

Surgery duration was defined as the interval from incision to completion of wound closure. It was obtained from the operative record duration field, with the source-verified corrections described below, and expressed per 60 min increment. For laboratory-window assignment, the operative end was derived as the recorded start timestamp plus this duration. The primary model additionally included preoperative hemoglobin concentration, the number of organs involved in the procedure, and the presence of upper-bowel and bladder surgery. The organ count comprised upper bowel, bladder, rectum or sigmoid colon, uterus, vagina, and ureter. The upper bowel was defined as the small bowel, appendix, cecum, and colon proximal to the rectosigmoid junction; rectosigmoid procedures were counted separately. Age at surgery was also described, together with the length of hospital stay, counted as inclusive calendar days from admission through discharge. Conversion to laparotomy was reported descriptively because the registry did not contain a standardized field for initial operative access.

We used three exploratory groupings to characterize the type and extent of surgery. First, rectosigmoid surgery, reported as rectosigmoid technique, was classified as no recorded discoid or segmental rectosigmoid resection (including shaving-only procedures), discoid resection without segmental resection, or any segmental resection. Second, a separate registry field indicated whether more than one full-thickness bowel resection had been performed. Among completed records, surgeries were classified as no resection when no full-thickness resection was recorded, one resection when a resection was recorded without the multiple-resection flag, and multiple resections when the flag was present. Neither the exact number of resections nor their anatomical distribution was recorded, and a blank field was treated as missing rather than as zero, so this variable was analyzed in complete cases only. Third, the organ count was categorized as no more than one, two, or three or more. Two further variables were handled separately: anastomotic height above the anal verge was modeled as a continuous variable and reported per 5 cm increment among patients undergoing segmental resection, and body mass index (BMI) was included only in sensitivity analyses.

An additional post hoc exploratory analysis combined resection multiplicity and organ count into three operative-burden strata. Low burden was defined as no recorded full-thickness bowel resection together with involvement of no more than one organ. High burden was defined as an explicitly recorded multiple bowel resection or involvement of three or more organs; all other surgeries were intermediate. For these strata, a blank multiplicity field was treated as no recorded multiple resection; it could prevent, but not create, high-burden assignment. A recorded full-thickness resection, identified from the separate discoid, segmental and other-site resection flags, was never overridden by a missing multiplicity field. Definitions were set before the composite group outcomes were calculated; none used fitted probabilities or outcome-derived cut points. The display rule was outcome-dependent; it is specified in the Supplementary Methods. These strata were intended as a descriptive clinical summary rather than a validated prediction rule.

### 2.5. Statistical Analysis

Continuous variables were summarized as medians and interquartile ranges, and categorical variables as *n*/*N* (%). Exact 95% confidence intervals (CIs) were calculated for proportions.

Multivariable logistic regression was used to examine factors associated with a hemoglobin decline of at least 3 g/dL. The outcome threshold was fixed before preliminary feasibility work. Surgery duration was selected as the principal exposure after that work, and the primary model was fixed before the final analysis. It included surgery duration, preoperative hemoglobin, organ count, upper-bowel surgery, and bladder surgery. Results are reported as adjusted odds ratios (ORs) with 95% CIs. Continuous hemoglobin decline was examined with the same covariates using linear regression with HC3 robust standard errors. Three implausibly short recorded durations had been checked against operative records by a clinician blinded to hemoglobin data and corrected before analysis; all models were subsequently refitted.

Because preoperative hemoglobin is part of the calculated decline, its apparent association with that decline can arise partly from arithmetic rather than biology [17,18]. We therefore repeated the binary and continuous analyses without preoperative hemoglobin. We also compared how well preoperative hemoglobin alone, the surgical variables alone, and both together discriminated between patients in whom the primary outcome did and did not occur. We summarized discrimination with the area under the receiver operating characteristic (ROC) curve (AUC) and overall prediction error with the Brier score, and we assessed calibration as the agreement between predicted and observed proportions [19,20]. Model performance was internally validated by patient-level bootstrap resampling; full specifications are provided in the Supplementary Methods. These association models were not intended for clinical risk prediction. Discrimination and calibration were reported to characterize model fit and the contribution of baseline hemoglobin, including its mathematical coupling with the endpoint.

Primary analyses included patients with both hemoglobin measurements. We summarized the pattern of hemoglobin availability and repeated the duration analysis using inverse-probability weighting, multiple imputation of the two hemoglobin components, and prespecified missing-not-at-random scenarios [21,22,23,24]. In separate sensitivity analyses we excluded surgeries involving the bladder, excluded patients with a recorded transfusion, allowed a nonlinear duration effect, replaced organ count with uterine and rectosigmoid indicators while retaining upper-bowel and bladder indicators, and added BMI (Table S2). Age and year of surgery were examined separately in post hoc sensitivity models. We also examined the last platelet count, activated partial thromboplastin time (APTT), and international normalized ratio (INR) recorded from 7 days before surgery until the recorded start of surgery. Each marker was added separately and all three were then added together to assess information beyond the primary model. None of these analyses replaced the primary model. Full specifications are provided in the Supplementary Methods.

Analyses of surgical type and extent were exploratory. Continuous decline was the main outcome, and the binary outcome was secondary. All models were adjusted for preoperative hemoglobin and were fitted both with and without surgery duration. For the post hoc operative-burden strata, we reported group-specific outcomes and an ordered trend across low, intermediate, and high burden. Adjustment for surgery duration was treated as an attenuation check because duration is itself one manifestation of operative burden. No outcome-based cut point, interaction, or confirmatory family was defined.

After peer review, additional post hoc analyses described the discharge day and sampling count, examined collinearity, and repeated the technique contrasts after excluding six known non-rectosigmoid resections from the reference group. A second sensitivity additionally excluded 15 reference-group patients with missing resection multiplicity. The original model and cohort were retained (Table S3). Bootstrap calibration slope and calibration-in-the-large were reported numerically (Table S4). Unadjusted duration estimates were also added for reporting completeness (Table S3, Panel F); these do not replace the prespecified adjusted estimates. Further post hoc analyses were specified in a dated amendment after early-discharge counts, and their event frequencies were known. Separate sensitivity analyses excluded patients discharged on or before postoperative day 3 and only those discharged before day 3. Another recalculated the nadir from the first recorded Hb of each calendar day within the unchanged postoperative window. Both binary and continuous models retained the original covariates (Table S3, Panel G).

Models and resampling were performed in R version 4.6.1 (R Foundation for Statistical Computing, Vienna, Austria). Figures were produced with ggplot2 version 4.0.3. Additional software details are provided in the Supplementary Methods. Missing-data reporting followed TARMOS. The TRIPOD and PROBAST checklists guided reporting of discrimination and calibration, although this study did not develop or validate a clinical prediction model [24,25,26].

### 2.6. Relationship to Previous Publications from the Center

Previous publications from our center examined bladder endometriosis, a modified segmental bowel-resection technique, and colorectal dehiscence or rectovaginal fistula [27,28,29]. One prior bladder cohort reported descriptive and subgroup perioperative hemoglobin changes. None of the prior publications assessed the full cohort using the present 3 g/dL endpoint, postoperative-day-0–3 window, and primary multivariable framework.

The reconstructed cohorts overlap: at least 402/424 patients appeared in one or more prior cohorts; the categories overlap, and the historical modified-resection count is a lower bound. The present analysis extends previous work from our center and is not based on an independent cohort. It does not re-evaluate the organ-specific complications reported in those studies. Table S1 distinguishes each previous study question, participant overlap, and the uncertainty of historical membership.

### 2.7. Use of Generative Artificial Intelligence

ChatGPT (Astra; OpenAI, San Francisco, CA, USA) and Claude (Opus 5.5; Anthropic, San Francisco, CA, USA) were used for English-language editing of the manuscript and for literature searches. No patient-level data were shared with these tools. The authors reviewed all outputs and take full responsibility for the manuscript.

## 3. Results

### 3.1. Hemoglobin Decline and Cohort Profile

**Figure 1:**
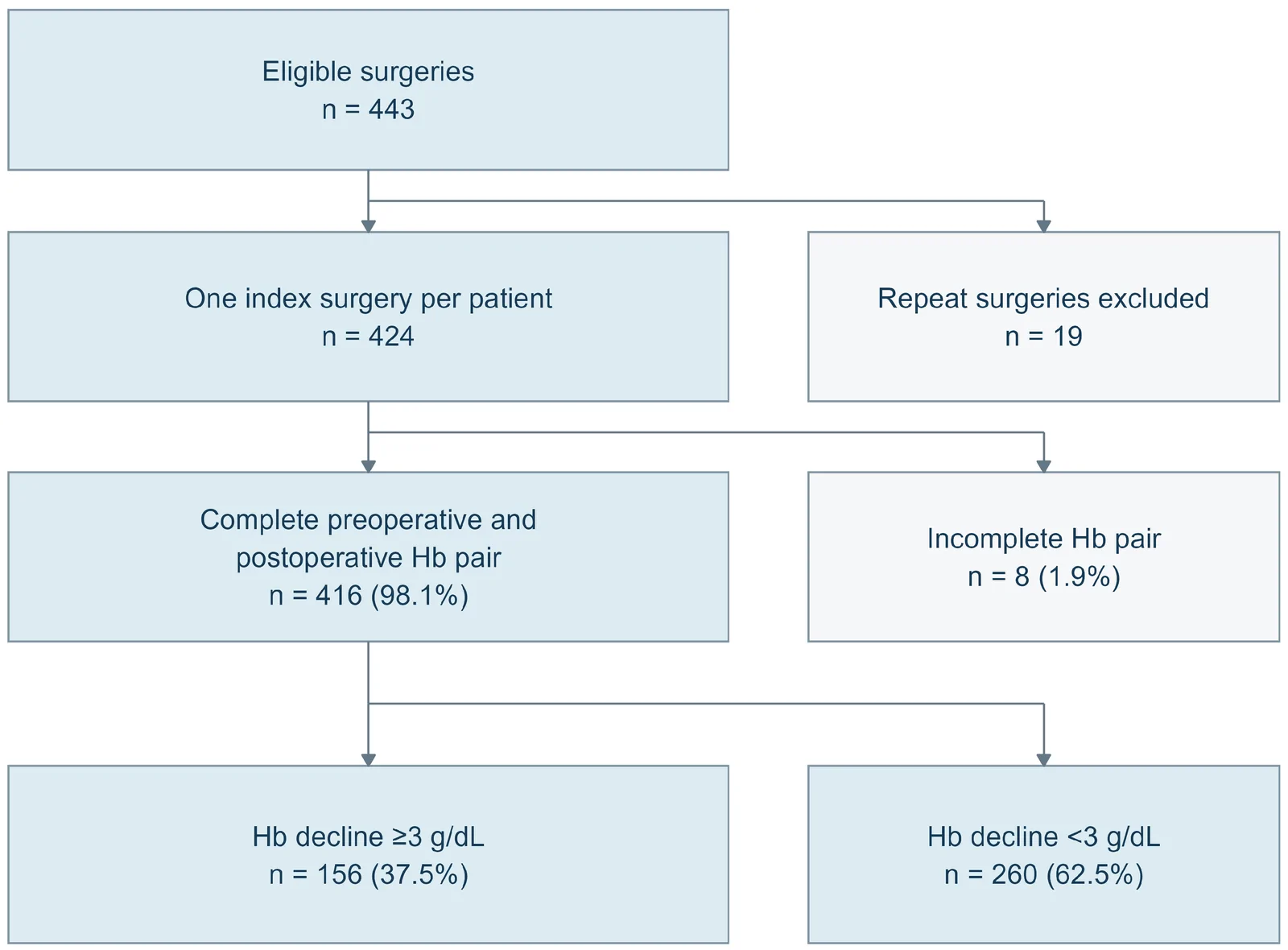
Study cohort. The diagram shows the number of eligible surgeries, exclusion of repeat surgeries, selection of one index surgery per patient, and availability of both preoperative and postoperative hemoglobin measurements. The final boxes give the number of patients with and without a hemoglobin decline of at least 3 g/dL.

The source cohort contained 443 eligible surgeries. After 19 repeat surgeries were excluded, 424 patients contributed one index surgery each (Figure 1). The median age was 38.3 years (IQR, 33.6–42.3), and the median length of hospital stay was 8 days (IQR, 8–9). The median interval from surgery to discharge was 5 days (IQR, 4–6), and the median preoperative stay was 2 days (IQR, 2–3). Among 419 patients with a preoperative measurement, 41 (9.8%) had hemoglobin below 12 g/dL and 152 (36.3%) below 13 g/dL. Recorded discharge occurred before postoperative day 3 in 19/424 patients (4.5%) and on day 3 in 37/424 (8.7%). All 19 discharged before day 3 had both Hb measurements. Among the 54 patients with both measurements who were discharged by postoperative day 3, 5 (9.3%) met the primary outcome (Table S3, Panel D).

A rectosigmoid procedure was performed in 383/424 patients (90.3%), uterine surgery in 273 (64.4%), and bladder surgery in 82 (19.3%). A ureteral procedure was performed in 63 patients (14.9%) and an upper-bowel procedure in 57 (13.4%) (Table 1).

Preoperative hemoglobin was available for 419/424 patients, a postoperative nadir for 421/424, and both measurements for 416/424 (98.1%). Among the 416 patients with both measurements, the median hemoglobin decline was 2.6 g/dL (IQR, 2.0–3.3). The primary outcome occurred in 156/416 patients (37.5%; exact 95% CI, 32.8–42.3%), and 42/416 (10.1%) had a decline of at least 4 g/dL. Median hemoglobin decreased from 13.3 g/dL (IQR, 12.6–14.0) before surgery to 10.6 g/dL (IQR, 9.7–11.4) at the recorded postoperative nadir. A recorded nadir below 9 g/dL occurred in 42/421 patients (10.0%), and 8/421 (1.9%) had a recorded nadir below 8 g/dL.

No recorded nadir occurred on postoperative day 0 (0/421). The lowest recorded postoperative value occurred on postoperative day 1 in 179/421 patients (42.5%), day 2 in 123 (29.2%), and day 3 in 119 (28.3%). This distribution describes the day of the lowest recorded value; it does not establish adherence to the daily sampling routine or within-patient hemoglobin trajectories. The mean number of recorded postoperative Hb measurements was 3.07 among patients meeting the primary outcome and 2.64 among those without it. These counts include all eligible measurements in the postoperative window, without a one-per-day limit. RBC transfusion was recorded in 42/424 patients (9.9%). Among the 42 transfused patients with both hemoglobin measurements, the primary outcome occurred in 29 (69.0%; Table 2). None of the eight patients with an incomplete Hb pair had a recorded transfusion.

### 3.2. Surgery Duration and Hemoglobin Decline

Longer surgery duration was associated with greater hemoglobin decline. In the primary model, each additional hour was associated with 61% higher odds of the primary outcome (OR, 1.61; 95% CI, 1.35–1.91). Removing preoperative hemoglobin did not materially weaken the association (OR, 1.50; 95% CI, 1.28–1.76).

**Figure 2:**
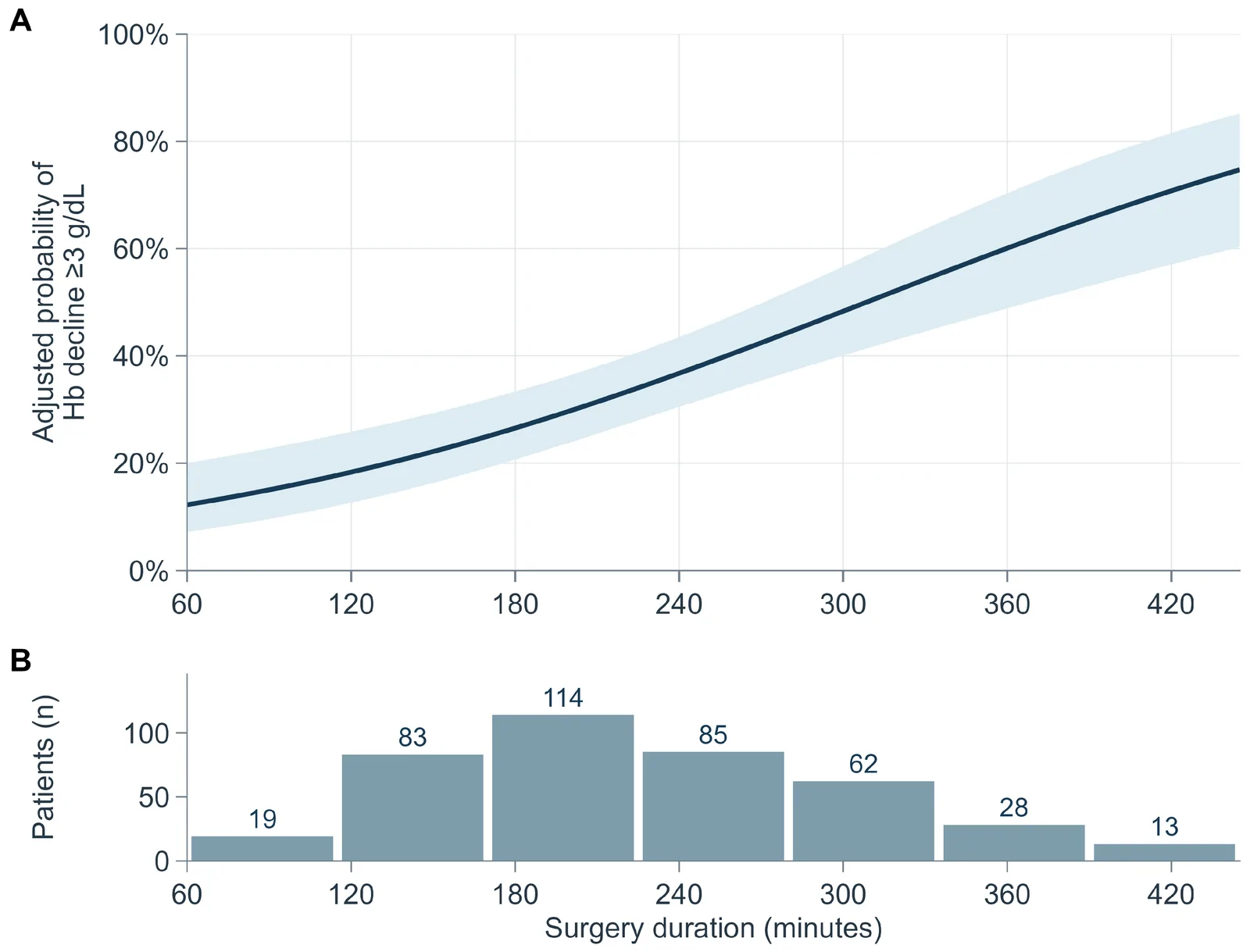
Adjusted probability of a hemoglobin decline of at least 3 g/dL according to surgery duration. The line shows estimates from the primary multivariable model for a preoperative hemoglobin concentration of 13.3 g/dL, involvement of two organs, and no upper-bowel or bladder procedure. The shaded area is the pointwise 95% confidence interval. For legibility, the display is limited to 60–445 min, a range that includes the surgery durations of 404/416 patients (97.1%); all 416 patients, including the 12 with durations beyond the displayed range, were retained in model estimation. Panel (**A**) shows the adjusted curve and confidence interval; panel (**B**) shows patient counts by surgery duration within the displayed range, using 55 min bins.

Each additional hour also corresponded to a 0.20 g/dL greater continuous decline, both with preoperative hemoglobin in the model (95% CI, 0.13–0.26) and without it (95% CI, 0.12–0.27). The interquartile range of surgery duration was 170 to 286 min. Across that interval, the modeled probability of the primary outcome increased from approximately 25% to 46% (Figure 2). Table 3 summarizes the binary and continuous duration estimates; the complete coefficient sets are given in Table S6.

Preoperative hemoglobin was strongly associated with crossing the fixed 3 g/dL threshold (OR per 1 g/dL, 2.32; 95% CI, 1.81–2.98). Because preoperative hemoglobin is part of the calculated decline, this coefficient reflects both the data and an arithmetic relationship. The association with surgery duration persisted in both models that excluded preoperative hemoglobin.

### 3.3. Clinically Recognizable Patterns of Surgical Extent

**Figure 3:**
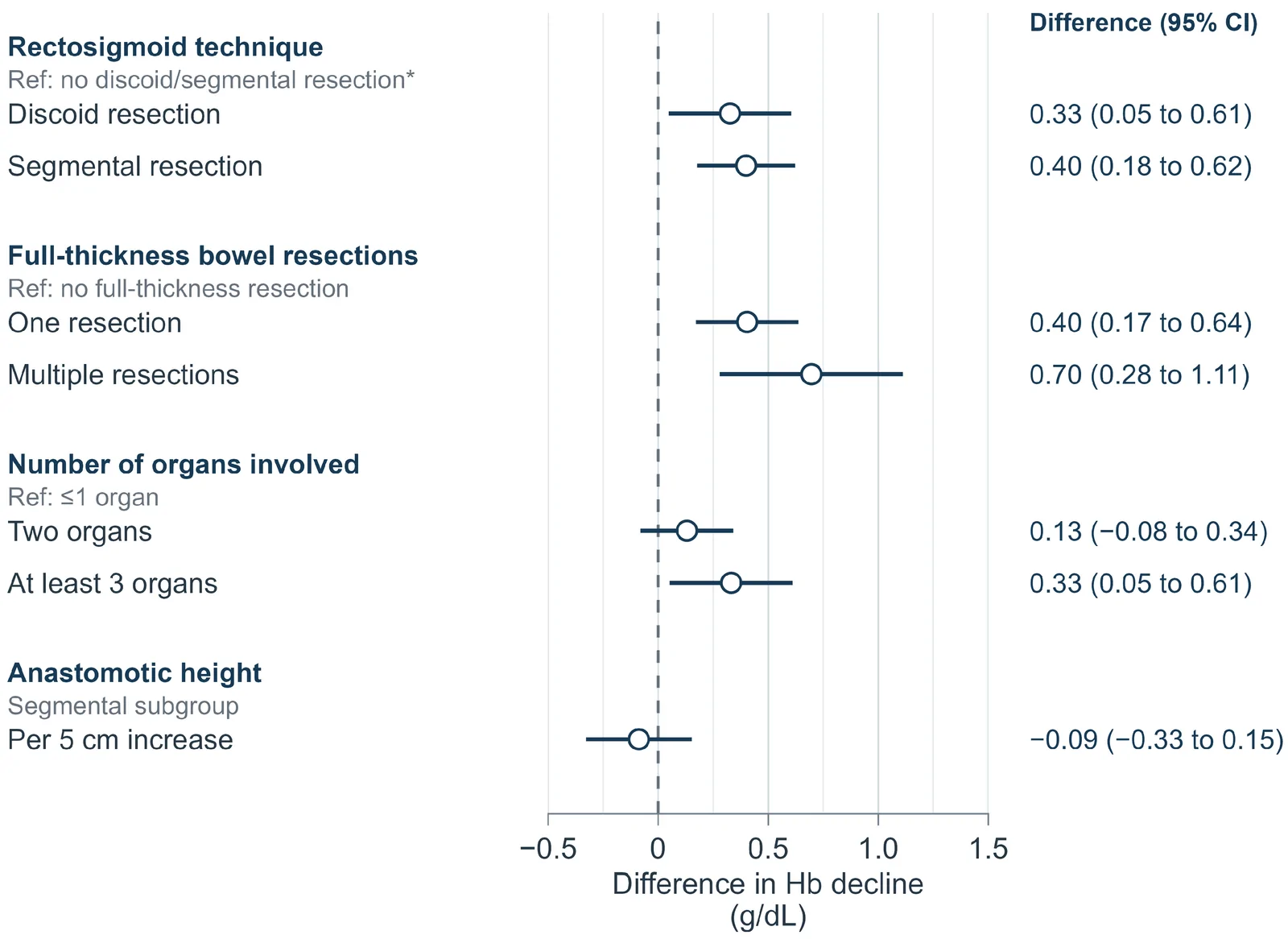
Associations of the type and extent of surgery with continuous hemoglobin decline. Circles show adjusted mean differences, and horizontal bars show 95% confidence intervals. Models were adjusted for preoperative hemoglobin and surgery duration. Reference groups were no recorded discoid or segmental rectosigmoid resection (including shaving-only procedures); no full-thickness bowel resection; and surgery involving one or fewer organs. The analysis included 416 patients for rectosigmoid technique and for the number of organs involved, 379 for resection multiplicity, and 240 for anastomotic height. Anastomotic height was analyzed per 5 cm increase. The dashed vertical line marks no difference (0 g/dL). * The rectosigmoid reference group includes shaving-only procedures, six full-thickness resections at other bowel sites, and 15 records with missing resection multiplicity; see Table 4 and Table S3.

Hemoglobin decline increased with the extent of rectosigmoid surgery (Figure 3 and Table 4). The primary outcome occurred in 17/83 patients (20.5%) in the reference group without recorded discoid or segmental rectosigmoid resection, 20/64 (31.2%) after discoid resection, and 119/269 (44.2%) after segmental resection (Table S7). With preoperative hemoglobin and duration in the model, the mean decline was 0.33 g/dL greater after discoid resection (95% CI, 0.05–0.61) than in the reference group. The corresponding difference after segmental resection was 0.40 g/dL (95% CI, 0.18–0.62).

A similar pattern was observed across the bowel-resection multiplicity categories. Among 379 patients with this field recorded, the outcome frequency was 9/62 (14.5%) with no full-thickness resection and 102/266 (38.3%) with one resection. It was 28/51 (54.9%) when multiple resections were performed during the same surgical session. In the same adjusted model, the mean decline was 0.40 g/dL greater after one resection (95% CI, 0.17–0.64). The corresponding difference after multiple resections was 0.70 g/dL (95% CI, 0.28–1.11). Corresponding ORs were 2.61 (95% CI, 1.13–6.04) and 3.46 (95% CI, 1.21–9.91). Because technique and multiplicity were separate registry fields, their denominators differed; details are provided in the Supplementary Methods. The endpoint occurred in 17/37 patients (45.9%) with missing multiplicity and in 139/379 (36.7%) with available multiplicity.

A comparable gradient was observed for the number of organs involved. The outcome frequency was 28/108 (25.9%) with no more than one organ involved, 76/204 (37.3%) with two organs, and 52/104 (50.0%) with at least three (Table S7). After adjustment for preoperative hemoglobin and duration, the mean decline was 0.13 g/dL greater for two organs than for no more than one (95% CI, −0.08 to 0.34). The corresponding difference for at least three organs was 0.33 g/dL (95% CI, 0.05–0.61).

No clear association was observed between anastomotic height and hemoglobin decline. Numeric anastomotic height was available in 240 of the 269 patients who underwent segmental resection. In this subgroup, the adjusted mean difference per 5 cm increase was −0.09 g/dL (95% CI, −0.33 to 0.15). Field availability is detailed in the Supplementary Methods.

Duration and organ count were correlated (Spearman ρ = 0.425). Design-based collinearity diagnostics and reference-group sensitivity analyses are reported in Table S3. After excluding the six known non-rectosigmoid resections, adjusted mean differences were 0.37 g/dL for discoid resection (95% CI, 0.10–0.65) and 0.45 g/dL for segmental resection (95% CI, 0.22–0.67). Restricting the reference to 62 patients with an explicit record of no full-thickness resection gave similar continuous estimates, while binary estimates were more sensitive to reference-group composition.

### 3.4. Descriptive Operative-Burden Strata

The post hoc operative-burden strata summarized these overlapping surgical features. The definitions were specified before group outcomes were calculated, but the strata were retained for presentation only after minimum-size, minimum-event, and monotonic-outcome display conditions were met. They are descriptive and require external validation. Low-, intermediate-, and high-burden strata included 44/416 (10.6%), 256/416 (61.5%), and 116/416 (27.9%) patients, respectively. The outcome frequency was 11/44 (25.0%), 85/256 (33.2%), and 60/116 (51.7%), and the mean hemoglobin decline was 2.12, 2.64, and 3.06 g/dL, respectively (Table 5).

After adjustment for preoperative hemoglobin and duration, mean decline was 0.30 g/dL greater per category increase (95% CI, 0.13–0.48). For the binary outcome, the corresponding estimate was imprecise (OR, 1.48; 95% CI, 0.96–2.28; Table S8).

### 3.5. Robustness of the Duration Finding

In post hoc discharge-restriction analyses, the duration OR per hour was 1.54 (95% CI, 1.28–1.84) after excluding discharge on or before day 3 (*n* = 362; 151 events), and 1.59 (95% CI, 1.33–1.90) after excluding discharge before day 3 (*n* = 397; 154 events). Using the first recorded Hb per calendar day yielded 153/416 events (36.8%), with 3 patients changing from event to non-event and a duration OR of 1.61 (95% CI, 1.35–1.91); continuous estimates for all three post hoc sensitivities are reported in Table S3, Panel G.

**Figure 4:**
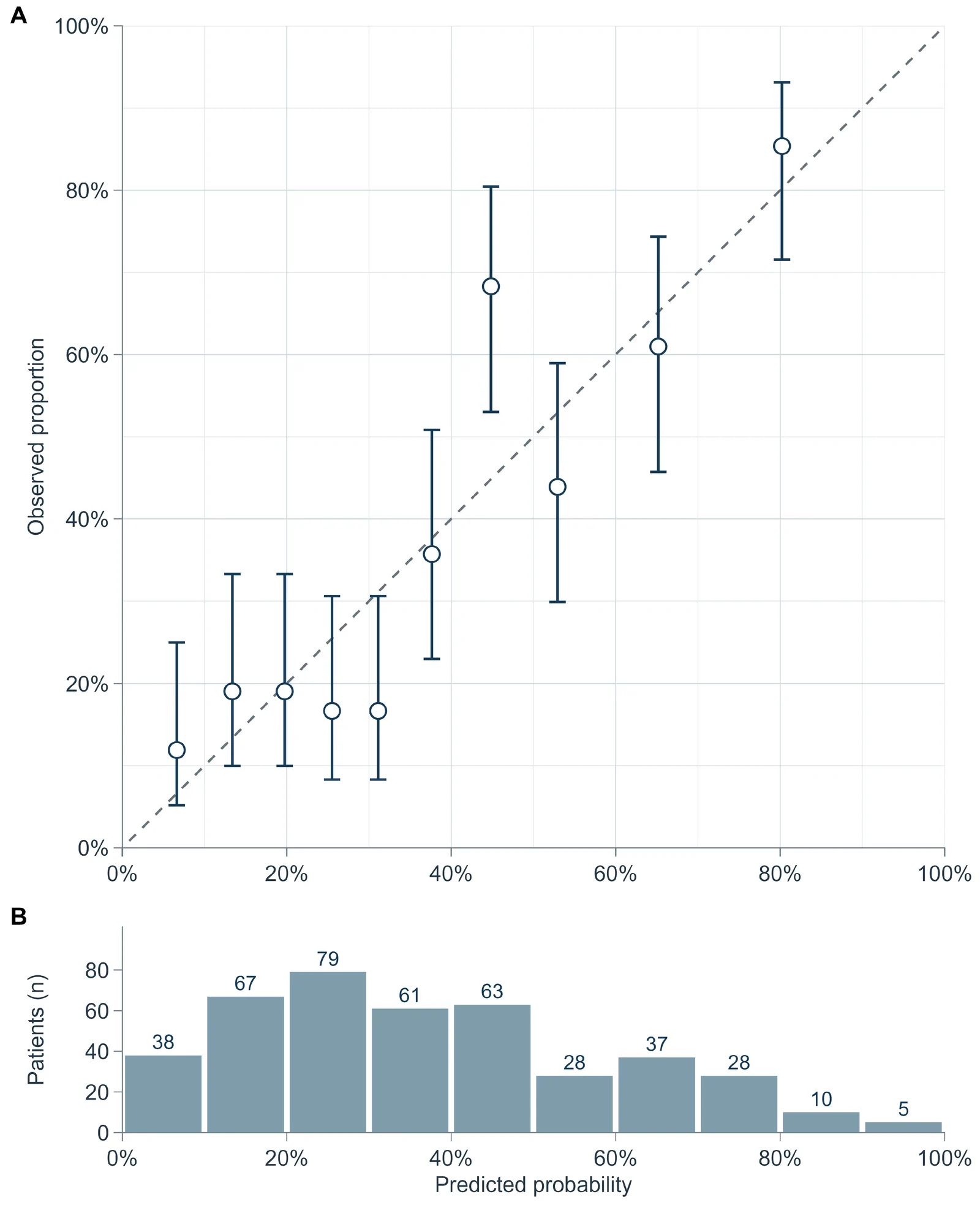
Agreement between predicted and observed proportions for the model including preoperative hemoglobin and surgical variables. Panel (**A**) shows apparent, in-sample calibration for Hb decline ≥ 3 g/dL by decile of predicted risk. Circles show observed proportions, vertical bars show Wilson 95% confidence intervals, and the diagonal represents ideal agreement. Decile counts and bootstrap summaries are in Table S4. The panels share the predicted-probability axis. Panel (**B**) uses 10-percentage-point bins and shows the predicted-risk distribution in the 416 patients with both preoperative and postoperative hemoglobin measurements.

Combining preoperative hemoglobin with surgical variables yielded an internally validated AUC of 0.760. Without preoperative hemoglobin, the surgical-variable model had an internally validated AUC of 0.684. On the apparent-performance scale used for paired comparisons, adding preoperative hemoglobin to the surgical model increased AUC by 0.074 (95% CI, 0.031–0.125). Adding surgical variables to the preoperative-hemoglobin model increased AUC by 0.085 (95% CI, 0.054–0.131). Both preoperative hemoglobin and the surgical variables contributed to discrimination (Table 3). Calibration within this cohort is shown in Figure 4. The bootstrap optimism-corrected calibration slope was 0.949 and calibration-in-the-large was +0.003; all 1000 resamples succeeded. These global summaries do not exclude local deviations in the decile plot (Table S4).

Alternative approaches to missing measurements produced comparable duration estimates. Inverse-probability weighting gave an OR of 1.61 (95% CI, 1.35–1.92). Multiple imputation gave an OR of 1.58 (95% CI, 1.33–1.88) and a continuous estimate of 0.19 g/dL per hour (95% CI, 0.13–0.25). Planned missing-not-at-random scenarios left the estimates essentially unchanged (Table S5).

Age was not clearly associated with either outcome (per 10 years: OR, 1.21; 95% CI, 0.82–1.79; continuous decline, 0.12 g/dL; 95% CI, −0.03 to 0.27). These intervals were wide and did not exclude a clinically relevant association. The restricted age distribution (IQR, 33.6–42.3 years in the full cohort) may have limited the ability to detect an age association. Across a broader adult age range (21–88 years), weak correlations of hemoglobin (r = 0.21) and platelet count (r = −0.22) with age were reported in 543 women after hysteroscopy for abnormal uterine bleeding; that population differs from ours in indication, operative intensity, and timing of blood sampling [30]. Including age did not materially change the duration estimate. Excluding surgeries involving the bladder yielded a consistent duration association (OR, 1.59; 95% CI, 1.31–1.94). There was no strong evidence of nonlinearity (*p* = 0.075). The per-hour estimate should be interpreted as an average association across the observed range. Year-of-surgery and other sensitivity analyses likewise preserved the duration association (Table S2).

In the 395 patients with BMI recorded, each 5 kg/m^2^ increase was associated with lower odds of the binary endpoint (OR, 0.55; 95% CI, 0.38–0.78), whereas the continuous estimate was smaller and imprecise (−0.11 g/dL; 95% CI, −0.23 to 0.01; Table S2).

Preoperative platelet count, APTT, and INR were available for 416, 415, and 413 patients with both hemoglobin measurements, respectively. None materially changed the duration estimate. Adding all three changed apparent AUC by 0.002 and provided no evidence of improved model fit (likelihood-ratio *p* = 0.822; Table S8). In the full cohort, median platelet count was 265 × 10^9^/L (IQR, 232–314.5; *n* = 419); 8/419 (1.9%) had counts <150 ×10^9^/L (Table S3).

## 4. Discussion

### 4.1. Main Findings

A hemoglobin decline of at least 3 g/dL occurred in 37.5% of patients with both measurements after surgery for deep endometriosis. Surgery duration remained associated with both binary and continuous decline when preoperative hemoglobin was removed from the model. Exploratory gradients were observed across rectosigmoid techniques, bowel-resection multiplicity, and the number of organs involved. The post hoc operative-burden strata also showed a descriptive gradient, but their display was outcome-dependent and the duration-adjusted binary trend was imprecise (OR, 1.48; 95% CI, 0.96–2.28). These exploratory patterns require external validation.

### 4.2. Comparison with Previous Studies

Earlier studies of deep endometriosis have generally reported measured blood loss, surgery duration, or postoperative complications rather than a defined hemoglobin-change outcome. Direct comparison with the 37.5% primary outcome frequency is therefore limited. These findings are nevertheless consistent with reports that extensive disease and bowel resection are accompanied by longer operating times and greater blood loss [8,9]. Specialist-center cohorts also show that morbidity varies with operative complexity [10]. We report a frequently observed, explicitly defined laboratory outcome that complements that clinical picture. The relative contributions of endpoint definition and case mix to differences between studies cannot be quantified from published aggregate data; neither should be assigned a numerical share.

### 4.3. Clinical Implications

Preoperative anemia and operative extent inform different steps in care. A lower preoperative hemoglobin increases the clinical impact of any given decline because the postoperative concentration will also be lower. Among patients with an available preoperative measurement, 9.8% had hemoglobin below 12 g/dL, compared with 23.9% using hematocrit < 36% in the ACS NSQIP uterine-surgery cohort [4]. The different procedures and definitions preclude a direct prevalence comparison. Using the ICCAMS threshold of Hb < 13 g/dL, 36.3% of our cohort met the criterion. Evaluation and treatment when indicated follow PBM guidance [2]; our Hb-decline model does not establish that anemia is protective or assess its causal impact.

Surgery duration and operative extent may help identify patients likely to experience a greater decline. The directly recorded surgical features—not the post hoc composite strata—provide the most transparent basis for future work. Their potential use in prioritizing postoperative monitoring requires prospective evaluation before they can define a clinical rule.

The lowest recorded postoperative hemoglobin value was on days 2–3 in 57.5% of the 421 patients with a postoperative measurement. Because adherence to routine sampling and within-patient day-1-versus-later comparisons were not evaluated, this distribution may reflect both postoperative trajectory and surveillance intensity. It should not be interpreted as evidence that routine testing on days 2–3 is superior to testing on day 1.

Hemoglobin decline reflects a combination of bleeding, hemodilution from fluid administration, transfusion, and the timing of sampling [31,32]. Accordingly, the observed gradients indicate operative burden rather than providing direct estimates of blood loss. The exploratory BMI association with crossing the fixed threshold, alongside a smaller continuous estimate, may reflect threshold behavior, body-size-related blood volume, or other confounding. Blood volume was not measured, and these data do not establish a protective BMI effect.

### 4.4. Interpretation of the Analyses Excluding Preoperative Hemoglobin

A fixed decline threshold is more readily crossed when preoperative hemoglobin is higher, because the preoperative value is part of the calculation [17,18]. This mathematical relationship helps explain why preoperative hemoglobin improved model performance and why its odds ratio should not be interpreted as a purely biological effect. Importantly, longer duration remained associated with both the binary outcome and continuous decline when preoperative hemoglobin was removed. Adding the surgical variables to the model containing preoperative hemoglobin improved discrimination. However, the surgical-variable model did not clearly outperform preoperative hemoglobin alone (difference in AUC, 0.011; 95% CI, −0.060 to 0.092).

### 4.5. Added Value and Future Research

Previous publications from our center focused on organ-specific technique or postoperative complications. This analysis addresses a distinct PBM question across the full cohort. It quantifies a perioperative hemoglobin outcome and shows that the duration association persisted when preoperative hemoglobin was omitted. Exploratory measures of operative extent showed gradients that require external validation.

Future studies should prospectively record measured blood loss, fluid balance, the cause and treatment of preoperative anemia, and transfusion timing, and should apply a standardized postoperative sampling schedule. External validation should then test whether the operative-burden strata improve monitoring decisions and whether continuous decline or a clinically validated threshold is the better endpoint.

### 4.6. Strengths and Limitations

This study included all eligible registry index surgeries, used clearly defined measurement windows, and captured both hemoglobin measurements in 98.1% of patients. After preliminary feasibility work, we fixed the primary model before the final analysis. The duration estimates were consistent across several approaches to the remaining missing measurements.

The retrospective, single-center design precludes control of variation in perioperative care and limits the applicability of the estimates to other settings. Rectosigmoid procedures were performed in 90.3% of patients, and the median hospital stay was 8 days; both features may differ in other centers. Surgeon and team effects, American Society of Anesthesiologists (ASA) physical status class, comorbidities, and anticoagulant exposure were not available for adjustment. Surgery duration should therefore be interpreted as a marker of operative complexity rather than a modifiable causal exposure. The cohort also overlaps with those of previous publications from our center and should be regarded as an extension rather than an independent validation cohort. Surgery duration was designated the principal exposure only after preliminary feasibility work; internal validation cannot account for that data-driven choice.

Quantitative measurements of blood loss and perioperative fluid volumes were not available, so bleeding could not be separated from hemodilution. Ferritin, transferrin saturation, and other measures of iron status were also unavailable; the cause of preoperative anemia could not be determined. Standardized fields for intravenous iron, tranexamic acid, and cell salvage were absent, precluding evaluation of these PBM measures. Transfusion status combined two discordant structured sources and was not clinically adjudicated; its timing relative to the recorded postoperative nadir was unavailable.

Eight patients lacked one or both hemoglobin measurements, and the missing-data analyses had little effect on the estimates. The missingness mechanism nevertheless cannot be established. Patients meeting the primary outcome had more recorded postoperative measurements than those without it (mean, 3.07 vs. 2.64); the reasons for this difference were not determined. A higher number of recorded measurements may have increased the chance of observing a lower value and created surveillance bias in this nadir-based outcome. Measurement count also increased modestly across duration quartiles (mean, 2.63 to 2.91; Spearman ρ = 0.136). Among patients with both measurements, 54/416 (13.0%) were discharged by day 3, including 19 before day 3, potentially truncating observation. A smaller Hb decline may itself have enabled earlier discharge. Calendar discharge dates cannot distinguish these mechanisms, and interdepartmental transfers were not separately adjudicated. The post hoc duration association was modestly attenuated after excluding discharge on or before day 3 and essentially unchanged with the first Hb per calendar day, but discharge restriction can introduce selection and daily-first sampling does not equalize monitored days or remove informative monitoring (Table S3, Panel G). Adherence to the daily sampling routine was not evaluated, and the timing of the lowest recorded value was described for the cohort as a whole rather than according to surgical extent. The observed distribution therefore cannot establish whether hemoglobin declined after day 1. Moreover, the lowest recorded value was on day 3 in 28.3% of patients, so a later true nadir may have been missed.

The 3 g/dL threshold is not a validated minimum clinically important difference. Resection multiplicity was unavailable for 37 patients with both hemoglobin measurements, and the surgical-extent analyses involved several exploratory comparisons without adjustment for multiple testing. Presentation of the post hoc operative-burden strata depended on minimum group sizes and monotonic outcome gradients. Missing multiplicity could prevent high-burden assignment, although recorded full-thickness resections still precluded low-burden classification; the direction of any resulting bias in the complete-case multiplicity estimates and in the burden gradient cannot be determined. These missing data and the outcome-dependent display rule limit the evidential weight of the strata, which require external validation. Finally, the narrow observed ranges of APTT and INR limit the hemostasis analysis; its findings do not apply to patients with clinically important coagulopathy. Thrombocytopenia was uncommon, but the platelet distribution does not by itself explain the neutral adjusted association or establish normal platelet function.

## 5. Conclusions

Longer surgery duration was associated with greater perioperative hemoglobin decline after surgery for deep endometriosis. Exploratory analyses also showed gradients across bowel-resection multiplicity and the number of organs involved, but these findings do not define a monitoring or prediction rule. These routinely available features warrant prospective evaluation as possible monitoring indicators, while lower preoperative hemoglobin indicates less reserve after a similar decline. The optimal timing of postoperative hemoglobin measurement cannot be determined from these retrospective data and should be evaluated prospectively with a standardized sampling schedule.

## Figures and Tables

**Table 1:** Patient and operative characteristics.

Characteristic	Full Cohort (*N* = 424)	Both Hemoglobin Measurements Available (*n* = 416)
Continuous characteristics, median (IQR)	—	—
Age at surgery, years	38.3 (33.6–42.3)	38.3 (33.5–42.2)
Surgery duration, min	218 (170–286)	220 (170–286)
Length of hospital stay, days	8 (8–9)	8 (8–9)
Body weight, kg	62.0 (56.0–70.0) [*n* = 381]	62.0 (56.0–70.0) [*n* = 374]
Height, cm	165 (161–168) [*n* = 379]	165 (161–168) [*n* = 372]
BMI, kg/m^2^	22.9 (20.9–25.5) [*n* = 402]	22.9 (20.9–25.5) [*n* = 395]
Clinical and operative characteristics, *n*/*N* (%)	—	—
Preoperative Hb < 12 g/dL	41/419 (9.8%)	41/416 (9.9%)
Preoperative Hb < 13 g/dL (additional descriptive threshold)	152/419 (36.3%)	152/416 (36.5%)
Organs involved: ≤1	111/424 (26.2%)	108/416 (26.0%)
Organs involved: 2	205/424 (48.3%)	204/416 (49.0%)
Organs involved: ≥3	108/424 (25.5%)	104/416 (25.0%)
Upper-bowel procedure	57/424 (13.4%)	56/416 (13.5%)
Bladder procedure	82/424 (19.3%)	79/416 (19.0%)
Rectosigmoid procedure	383/424 (90.3%)	376/416 (90.4%)
Uterine procedure	273/424 (64.4%)	267/416 (64.2%)
Vaginal procedure	12/424 (2.8%)	12/416 (2.9%)
Ureteral procedure	63/424 (14.9%)	63/416 (15.1%)
Conversion to laparotomy	11/424 (2.6%)	11/416 (2.6%)

Values are median (IQR) or *n*/*N* (%). A bracketed *n* denotes the number of available observations for anthropometric variables. BMI was independently recorded in the patient record and was not recomputed from the displayed weight and height fields. Eight patients lacked one or both hemoglobin measurements; because this group is too small to describe by individual characteristics, Table S5 reports only the pattern of component availability; Tables S2 and S3 additionally report recorded conversion, transfusion and discharge timing by completeness. No between-group hypothesis tests were performed. Hb, hemoglobin; IQR, interquartile range; BMI, body mass index.

**Table 2:** Perioperative hemoglobin and transfusion outcomes.

Outcome	Estimate	95% CI
Primary outcome	—	—
Hb decline ≥ 3 g/dL	156/416 (37.5%)	32.8–42.3%
Secondary hemoglobin outcomes	—	—
Continuous Hb decline, g/dL	2.6 (2.0–3.3)	—
Hb decline ≥ 4 g/dL	42/416 (10.1%)	7.4–13.4%
Recorded postoperative nadir < 9 g/dL	42/421 (10.0%)	7.3–13.2%
Recorded postoperative nadir < 8 g/dL	8/421 (1.9%)	0.8–3.7%
Transfusion outcome	—	—
Red blood cell (RBC) transfusion during the index hospitalization	42/424 (9.9%)	7.2–13.2%
Hb decline ≥ 3 g/dL among transfused patients with both hemoglobin measurements	29/42 (69.0%)	52.9–82.4%

Values are *n*/*N* (%) unless otherwise stated. Exact (Clopper–Pearson) 95% confidence intervals are shown for proportions; continuous Hb decline is summarized as median (IQR). Hb, hemoglobin; IQR, interquartile range; CI, confidence interval; RBC, red blood cell.

**Table 3:** Surgery duration and model performance with and without preoperative hemoglobin.

Panel A. Duration association across binary and continuous outcomes				
Outcome and Model		Patients, *n*	Duration Estimate per 60 min (95% CI)	*p* Value
Hb decline ≥ 3 g/dL, primary model		416	OR 1.61 (1.35 to 1.91)	<0.001
Hb decline ≥ 3 g/dL, model without preoperative Hb		416	OR 1.50 (1.28 to 1.76)	<0.001
Continuous Hb decline, model with preoperative Hb		416	0.20 (0.13 to 0.26) g/dL	<0.001
Continuous Hb decline, model without preoperative Hb		416	0.20 (0.12 to 0.27) g/dL	<0.001
Recorded postoperative Hb nadir, adjusted for preoperative Hb, organ count, upper-bowel surgery, and bladder surgery		416	−0.20 (−0.26 to −0.13) g/dL	<0.001
Panel B. Apparent and internally validated model performance				
Model	Apparent AUC	Internally Validated AUC	Apparent Brier Score	Internally Validated Brier Score
Preoperative Hb only	0.684	0.684	0.211	0.214
Surgical variables without preoperative Hb	0.696	0.684	0.212	0.217
Preoperative Hb plus surgical variables	0.770	0.760	0.182	0.188
Panel C. Bootstrap differences in apparent AUC				
Contrast			Δ AUC (95% Bootstrap CI)	
Preoperative Hb added to the surgical-variable model			0.074 (0.031 to 0.125)	
Surgical variables added to the preoperative-Hb model			0.085 (0.054 to 0.131)	
Surgical-variable model versus preoperative Hb alone			0.011 (−0.060 to 0.092)	

The preoperative-Hb model contains preoperative hemoglobin only. The surgical-variable model contains surgery duration, organ count, upper-bowel surgery, and bladder surgery. The combined model contains both sets of variables. The recorded-postoperative-nadir row is an algebraic re-expression of the continuous-decline row, because the nadir equals preoperative hemoglobin minus the decline in a model that already adjusts for preoperative hemoglobin; it is shown for completeness and is not an independent confirmation. The three logistic models in Panel B were internally validated with 1000 bootstrap resamples; internally validated values equal apparent performance minus the average bootstrap optimism. Point estimates in Panel C are differences in the unrounded apparent AUCs, not bootstrap means or medians; subtracting the rounded Panel B entries can differ by 0.001. Percentile 95% CIs used 2000 paired bootstrap resamples. Hb, hemoglobin; OR, odds ratio; CI, confidence interval; AUC, area under the receiver operating characteristic curve.

**Table 4:** Exploratory associations of the type and extent of surgery with hemoglobin decline.

Contrast	Patients, *n*	Adjusted Mean Difference in Hb Decline, g/dL (95% CI)	Adjusted OR for Hb Decline ≥ 3 g/dL (95% CI)
Rectosigmoid technique (reference: no recorded discoid/segmental rectosigmoid resection, including shaving-only procedures)	—	—	—
Discoid resection	416	0.33 (0.05 to 0.61)	1.66 (0.73 to 3.74)
Segmental resection	416	0.40 (0.18 to 0.62)	2.06 (1.06 to 4.00)
Number of full-thickness bowel resections (reference: no resection)	—	—	—
One resection	379	0.40 (0.17 to 0.64)	2.61 (1.13 to 6.04)
Multiple resections	379	0.70 (0.28 to 1.11)	3.46 (1.21 to 9.91)
Number of organs involved (reference: ≤1 organ)	—	—	—
Two organs	416	0.13 (−0.08 to 0.34)	1.47 (0.83 to 2.58)
≥3 organs	416	0.33 (0.05 to 0.61)	1.89 (0.94 to 3.79)
Anastomotic height above the anal verge	—	—	—
Per 5 cm increase	240	−0.09 (−0.33 to 0.15)	0.87 (0.46 to 1.66)

All models are adjusted for preoperative Hb and surgery duration. Continuous models use HC3 robust standard errors. Reference categories are given in parentheses in the group-heading rows. Rectosigmoid technique denotes the rectosigmoid technique group defined in the Methods. The 83 reference-group patients included 62 with an explicit record of no full-thickness bowel resection, 6 with full-thickness resection at a non-rectosigmoid site, and 15 with missing resection multiplicity. The reference group is therefore not free of bowel resection. Both post hoc sensitivity analyses addressing its composition are reported in Table S3. Hb, hemoglobin; OR, odds ratio; CI, confidence interval.

**Table 5:** Post hoc descriptive operative-burden strata and hemoglobin outcomes.

Operative-Burden Stratum	Patients, *n*/*N* (%)	Hb Decline ≥ 3 g/dL, *n*/*N* (%; Exact 95% CI)	Hb Decline, Mean (95% CI), g/dL	Hb Decline, Median (IQR), g/dL
Low burden	44/416 (10.6%)	11/44 (25.0%; 13.2–40.3%)	2.12 (1.80 to 2.45)	2.0 (1.2–3.0)
Intermediate burden	256/416 (61.5%)	85/256 (33.2%; 27.5–39.3%)	2.64 (2.53 to 2.75)	2.5 (2.0–3.2)
High burden	116/416 (27.9%)	60/116 (51.7%; 42.3–61.1%)	3.06 (2.84 to 3.28)	3.0 (2.2–3.8)

Low burden was defined as no recorded full-thickness bowel resection and no more than one organ involved. High burden was defined as an explicitly recorded multiple bowel resection or at least three organs involved; all remaining surgeries were intermediate. When a surgery met both the low-burden and the high-burden definition, the high-burden definition took precedence. A blank resection-multiplicity field was treated as no recorded multiple resection; it could therefore prevent, but never create, assignment to the high-burden group. The low-burden criterion used the all-site full-thickness-resection indicator derived from recorded discoid, rectosigmoid segmental and other bowel-site resection flags, independently of the multiplicity field. Missing multiplicity did not erase a recorded full-thickness resection. A missing multiple-resection flag could therefore leave a patient in the intermediate or low group according to the other procedure and organ indicators. The definitions were specified before group outcomes were calculated, but the strata were retained for presentation only after minimum-size, minimum-event, and monotonic-outcome display conditions were met. This display rule was outcome-dependent. The strata are descriptive and require external validation. Confidence intervals for proportions are exact (Clopper–Pearson). Hb, hemoglobin; IQR, interquartile range; CI, confidence interval.

## Data Availability

Patient-level data are not publicly available because of privacy and institutional governance restrictions. Aggregate data and analytical code may be made available by the corresponding author upon reasonable request, subject to institutional approval.

## Supplementary Materials

The following supporting information can be downloaded at: https://www.mdpi.com/article/10.3390/jcm15208084/s1, Supplementary Methods provide full specifications for resampling, missing data, and exploratory analyses. Table S1 maps previous cohorts; Table S2 reports sensitivity analyses; Table S3, additional post hoc analyses added during peer review; Table S4, decile and bootstrap calibration summaries; Table S5, component-level missingness, multiple imputation, and missing-not-at-random analyses; Table S6, complete coefficient sets for the binary and continuous duration models; Table S7, surgical-group profiles and additional model information; and Table S8, post hoc operative-burden and preoperative hemostatic-marker analyses. Completed STROBE and RECORD checklists are supplied separately.

## Abbreviations

APTT, activated partial thromboplastin time; AUC, area under the receiver operating characteristic curve; BMI, body mass index; CI, confidence interval; Hb, hemoglobin; HC3, heteroskedasticity-consistent covariance estimator; INR, international normalized ratio; IQR, interquartile range; OR, odds ratio; PBM, patient blood management; RBC, red blood cell.
