# Supplementary material for: Perioperative Hemoglobin Decline After Deep Endometriosis Surgery: Association with Surgery Duration and Exploratory Analyses of Operative Extent
Source: J Clin Med. 2026 Oct 18;15(20):8084. doi: 10.3390/jcm15208084 (PMC13706007; doi:10.3390/jcm15208084)
Supplement: Supplementary file 1 [file jcm-15-08084-s001.zip › jcm-4543573-checklist.pdf]

STROBE and RECORD reporting checklists

Manuscript jcm-4543573 — revised manuscript (Round 1)

Items below are paraphrased from the official STROBE cohort and RECORD checklists. Locations refer to section and table numbers in the revised manuscript.

STROBE cohort checklist

Item	Reporting topic	Location or explanation
1a	Study design	Abstract Methods: single-center retrospective study
1b	Balanced abstract	Abstract Methods, Results and Conclusions
2	Scientific rationale	Section 1
3	Objectives and chronology	Section 1 final aim; Section 2.5; Supplementary Methods
4	Study design	Section 2.1
5	Setting and dates	Section 2.1, including usual admission timing and individualized discharge criteria; observation windows in 2.3
6a	Eligibility, selection and follow-up	Sections 2.2–2.3; Figure 1
6b	Matching	Not applicable: unmatched cohort
7	Variables and definitions	Sections 2.3–2.4; Supplementary Methods. Duration is defined as incision to completion of wound closure.
8	Sources and measurements	Sections 2.1–2.4; Supplementary Methods. Usual admission timing is described in Section 2.1; routine daily blood counts starting on postoperative day 1 are described in Section 2.3.
9	Bias	Sections 2.5, 2.6 and 4.6; Tables S3 and S7
10	Study size	Section 2.2: all eligible index surgeries; no a priori sample-size calculation
11	Quantitative variables	Sections 2.3–2.5; Tables 1, 4, 5; Supplementary Methods
12a	Statistical methods and adjustment	Section 2.5; Supplementary Methods
12b	Subgroups and interactions	Section 2.5; Supplementary Methods: no interactions; exploratory extent categories
12c	Missing data	Section 2.5; Supplementary Methods; Table S3
12d	Incomplete follow-up	Hb availability in Section 3.1; discharge-window limitation in 4.6 and Table S7; no long-term outcome follow-up
12e	Sensitivity analyses	Sections 2.5 and 3.5; Tables S1, S3 and S7
13a	Numbers at each stage	Section 3.1; Figure 1, starting at eligible linked operations; earlier exclusions not reconstructed (2.2)
13b	Reasons for exclusion	Section 2.2; Figure 1 (repeat surgeries, unavailable pairs); earlier linkage/source-completeness counts not reconstructed
13c	Flow diagram	Figure 1

Item	Reporting topic	Location or explanation
14a	Descriptive characteristics	Table 1; Table S2 surgical subgroups
14b	Missingness by variable	Tables 1, S1–S3 and S5; Sections 3.1, 3.3, 3.5
14c	Follow-up time	Sections 2.3 and 3.1; Table S7 discharge timing; laboratory endpoint through day 3
15	Outcome frequencies	Section 3.1; Table 2; surgical groups in Table S2
16a	Unadjusted and adjusted estimates	Unadjusted duration estimates in Table S7 Panel F; unadjusted surgical-group frequencies in Table S2; adjusted primary/extent models in Tables 3–4 and S4
16b	Category boundaries	Section 2.4; Tables 4–5 and S7
16c	Absolute risks	Endpoint proportions in Tables 2 and S2; Figure 2 conditional probability, not a validated clinical prediction rule
17	Other analyses	Sections 3.3–3.5; Tables S1–S8
18	Key results	Section 4.1
19	Limitations	Section 4.6; uncertainty in direction of missingness and ascertainment bias is explicit
20	Interpretation	Sections 4.2–4.5 and 5
21	Generalizability	Sections 4.5–4.6 and 5
22	Funding and funder role	Funding statement: no external research funding; APC support separately identified

RECORD extension

Item	Reporting topic	Location or explanation
1.1	Data type and database	Abstract Methods: institutional registry and laboratory records
1.2	Region and timeframe	Abstract Methods: Opole, Poland, 2020–2025
1.3	Data linkage in abstract	Abstract Methods explicitly states linked registry and laboratory records
6.1	Selection algorithm	Section 2.2; Supplementary Methods linkage/selection details
6.2	Validation of selection algorithm	Supplementary Methods: no external validation study of selection codes; local linkage/invariant checks and clinical correction described
6.3	Linkage flow	Figure 1 and Section 3.1: 443 eligible linked operations, 424 index patients, 419 baseline Hb, 421 nadirs, 416 pairs. Earlier failed-linkage/source-completeness counts were not reconstructed (Section 2.2).
7.1	Codes and classification algorithms	Supplementary Methods specify the transfusion ascertainment algorithm and ICD-9 99.04; Sections 2.3–2.4 describe the clinical variables and surgical category hierarchy
12.1	Investigators' database access	Supplementary Methods; Data Availability Statement
12.2	Data cleaning	Supplementary Methods laboratory reconciliation and blinded duration correction; Section 2.5

Item	Reporting topic	Location or explanation
12.3	Linkage method and quality	Supplementary Methods linkage/selection details; local deterministic linkage, aggregate completeness and one-patient/one-operation checks
13.1	Selection and linkage restrictions	Sections 2.2 and 3.1; Figure 1; Table S3
19.1	Routinely collected data limitations	Section 4.6: missingness, surveillance, unmeasured confounding, source-code/transfusion uncertainty, post-feasibility choice
22.1	Access to protocol/data/code	Supplementary Methods; Data Availability Statement: patient data restricted, aggregate data/code on reasonable request subject to institutional approval

Sources: https://www.static.strobe-statement.org/checklists/ and https://www.record-statement.org/Files/checklist/RECORD%20Checklist.pdf. Original forms retained in the project source archive.
