# Supplementary material for: Perioperative Hemoglobin Decline After Deep Endometriosis Surgery: Association with Surgery Duration and Exploratory Analyses of Operative Extent
Source: J Clin Med. 2026 Oct 18;15(20):8084. doi: 10.3390/jcm15208084 (PMC13706007; doi:10.3390/jcm15208084)
Supplement: Supplementary file 1 [file jcm-15-08084-s001.zip › jcm-4543573-supplementary.pdf]

Supplementary material

Manuscript jcm-4543573

Supplementary Methods

HC1 and HC3 denote heteroskedasticity-consistent covariance estimators, and ROC denotes the receiver operating characteristic curve.

Laboratory-data acquisition and reconciliation

The departmental routine was to obtain complete blood counts daily starting on postoperative day 1. The retrospective analysis used the recorded measurements available within the predefined windows; adherence to the daily sampling routine was not evaluated.

The institutional registry was linked locally to structured laboratory records. Legacy records with dates absent from the portal view were reconciled against dates stored in source PDF reports and order metadata. The final analysis used the completed extraction. BMI was taken from its separately recorded numeric patient field, rather than recalculated from the available weight and height fields; completeness can therefore differ. Aggregate quality checks identified 69 newly selected preoperative values, 21 newly selected postoperative nadirs, and 14 earlier nadir selections that were superseded. A separate manual paper-chart import contributed no hemoglobin values. For each patient, the selection rules described in the main manuscript were applied after this reconciliation.

Comparative models and internal validation

The 3-g/dL outcome threshold was fixed before preliminary feasibility work. Surgery duration was selected as the principal exposure after that work, and the primary model specification was fixed before the final analysis. The primary complete-case logistic model included surgery duration, preoperative hemoglobin, number of organs involved in the procedure, upper-bowel surgery, and bladder surgery. All variables were entered simultaneously; neither an automated selection procedure nor an outcome-based entry criterion was used. The same covariates were used in a linear model of continuous hemoglobin decline, fitted with HC3 robust standard errors.

To assess mathematical coupling between preoperative hemoglobin and hemoglobin change, three logistic models were compared in the same 416 patients: preoperative hemoglobin alone; surgical variables without preoperative hemoglobin (duration, organ count, upper-bowel surgery, and bladder surgery); and both sets of variables together. Apparent AUC and Brier score were estimated for each model. Internal validation used 1000 patient-level bootstrap samples with the same model specification in every resample. In each sample, patients were drawn with replacement until a dataset of the same size was obtained. The model was refitted in each resample, the optimism of that refit was estimated, and the mean optimism across resamples was subtracted from the apparent performance of the original model. Paired nonparametric bootstrap resampling with 2000 samples estimated AUC differences between each pair of models. Continuous decline was also modeled without preoperative hemoglobin. For completeness, postoperative nadir was modeled with adjustment for preoperative hemoglobin; because decline equals baseline minus nadir, this was treated as an algebraic re-expression rather than independent corroboration.

Calibration of the combined model was examined in deciles of predicted risk. Internal validation used bootstrap resampling in all 416 patients included in the model; no random training-test split was applied.

Missing measurements

We reported component-level hemoglobin availability, recorded transfusion and conversion frequencies in the incomplete-pair group, and discharge timing by pair completeness. Standardized differences for weighting diagnostics compared the complete-pair group with the full cohort, as specified below. Inverse-probability weighting (IPW) modeled the probability that both hemoglobin measurements were available as a function of surgery duration, the number of organs involved, and upper-bowel, bladder, and uterine procedures. Stabilized weights were truncated at the 1st and 99th percentiles, and the weighted outcome model used HC1 sandwich variance. The confidence interval for the weighted duration estimate was conditional on the estimated weights. The balance diagnostic in Table S2 Panel F compares complete cases, with and without weighting, against the full 424-patient cohort, not against the eight incomplete cases.

Multiple imputation targeted the preoperative and postoperative hemoglobin components, not the derived change score. A total of 50 datasets were imputed by predictive mean matching with five donors, using 20 iterations of the chained-equations algorithm. The predictor matrix included both hemoglobin components, surgery duration, five non-lower-bowel organ indicators, three mutually exclusive lower-bowel technique indicators, conversion, and recorded transfusion. The organ-count variable and the redundant general lower-bowel indicator were omitted from the imputation equations to avoid rank deficiency; their observed values were used wherever those variables entered an outcome model. Change scores and binary outcomes were derived within each completed dataset, and estimates were combined with Rubin's rules.

A pattern-mixture analysis examined departures from the missing-at-random (MAR) assumption; scenarios that depart from it are described as missing not at random (MNAR). Imputed preoperative hemoglobin was shifted by -0.5, 0, or +0.5 g/dL, and imputed postoperative nadir by 0, -0.5, -1.0, or -1.5 g/dL, giving 12 prespecified scenarios. These analyses did not replace the complete-case primary analysis.

As a descriptive surveillance diagnostic, postoperative measurement intensity was compared by primary-outcome status (mean number of measurements, 3.07 with the outcome vs. 2.64 without it). This comparison was not used as a covariate or post-exposure adjustment; it was reported only to characterize potential ascertainment bias in the nadir-based outcome.

Age at surgery was derived from the recorded surgery date and the patient's date of birth. Length of hospital stay was counted as inclusive calendar days from admission through discharge. Preoperative anemia was defined as a hemoglobin concentration below 12 g/dL among patients with an available preoperative value. After peer review, Hb <13 g/dL was additionally reported with the same denominator. These variables were reported descriptively and did not enter the primary model.

Age and year of surgery were each added separately to the binary and continuous primary models in post hoc sensitivity analyses. Age was scaled per 10 years and year of surgery per one-year increase. These analyses assessed whether the duration estimate changed materially; they did not replace the primary model.

Other sensitivity and exploratory analyses

Transfusion was considered present if recorded in the dedicated transfusion table or with ICD-9 procedure code 99.04. Discordant records were not clinically adjudicated, and transfusion timing relative to the recorded postoperative nadir was unavailable.

Separate sensitivity analyses excluded surgeries involving the bladder; added a quadratic duration term; replaced the organ count with separate uterine and rectosigmoid indicators; added body mass index (BMI); and excluded patients with a recorded transfusion during the index hospitalization. Conversion to laparotomy was reported descriptively only. Three recorded surgery durations were implausibly short. They were checked against the operative records by a clinician who was blinded to the hemoglobin data and were corrected before analysis; all models were then refitted. After correction, no recorded surgery duration was below 45 minutes.

In the analyses of surgical type and extent, continuous hemoglobin decline was the main outcome and the binary outcome was secondary. All models were adjusted for preoperative hemoglobin and were fitted both with and without surgery duration. Logistic regression was used for the binary outcome and linear regression with HC3 robust standard errors for continuous decline. Multi-category variables were tested with overall likelihood-ratio tests or HC3-based Wald tests. Changes in AUC, Brier score, and R^2 were interpreted descriptively and were not formally tested. Missing resection multiplicity was not imputed. No interactions or anastomotic-height cut points were examined, and no confirmatory family was defined.

The bowel-technique variable and the resection-multiplicity field were separate source variables and therefore had different denominators. A separate registry field indicated whether more than one full-thickness bowel resection had been performed. Among completed records, surgeries were classified as no resection when no full-thickness resection was recorded, one resection when a resection was recorded without the multiple-resection flag, and multiple resections when the flag was present. The field did not provide the exact number or anatomical distribution of resections. Of the 83 patients assigned to the reference technique group, 62 were explicitly recorded as having had no full-thickness resection; 6 had a full-thickness resection at a bowel site other than the rectosigmoid, so the resection was captured by the multiplicity field but not by the technique variable; and in 15 the multiplicity field was blank. A blank field was treated as missing rather than as zero. Numeric anastomotic height was available for 240/269 paired segmental-resection cases (89.2%) and was unavailable for the remaining 29.

Post hoc operative-burden strata

The low-, intermediate-, and high-burden definitions were set before the composite group outcomes were calculated. Low burden required no recorded full-thickness bowel resection and involvement of no more than one organ. High burden required an explicitly recorded multiple bowel resection or involvement of three or more organs; when a surgery met both the low-burden and the high-burden definition, the high-burden definition took precedence. All remaining surgeries were intermediate. For these strata, a blank multiplicity field was handled as no recorded multiple resection and could therefore prevent, but never create, assignment to the high-burden group.

The definitions did not use hemoglobin outcomes, fitted probabilities, ROC cut points, or p values. The strata were retained for presentation only after the following display conditions were met: at least 40 surgeries and at least 10 events per stratum, with monotonic increases in both event frequency and mean decline. This display rule is outcome-dependent and is reported here for transparency. Group-specific event frequencies used exact binomial 95% confidence intervals. Continuous decline was summarized with means and 95% confidence intervals and medians with interquartile ranges. An ordered term coding low, intermediate, and high burden as 0, 1, and 2 was examined without adjustment, after adjustment for preoperative hemoglobin, and after additional adjustment for surgery duration. The duration-adjusted result was interpreted as an attenuation check because duration is one manifestation of operative burden. The low-burden criterion used the all-site full-thickness-resection indicator derived from recorded discoid, rectosigmoid segmental and other bowel-site resection flags, independently of the multiplicity field. Missing multiplicity did not erase a recorded full-thickness resection. A missing multiple-resection flag could therefore leave a patient in the intermediate or low group according to the other procedure and organ indicators. These strata are descriptive and not a validated prediction rule.

Preoperative platelet count and coagulation tests

The last platelet count, activated partial thromboplastin time (APTT), and international normalized ratio (INR) recorded from 7 days before surgery until the recorded start of surgery were examined in post hoc exploratory analyses. Each marker was added separately to the primary model, in the subset of patients for whom that marker was available, and was also examined with continuous hemoglobin decline. A combined block containing all three markers was compared with the reference model using a likelihood-ratio test, change in apparent AUC, adjusted R^2, and root mean squared error. Effects were expressed per 50 × 10^9/L for platelet count, per 5 s for APTT, and per 0.1 for INR. These analyses assessed whether these markers added information about hemoglobin decline beyond the primary model; they did not evaluate the clinical indications for coagulation testing.

Data linkage and reproducibility

Authorized local access was used to link the operative registry, hospital records and laboratory results. Registry operations were matched deterministically to operative records using the internal patient identifier and operation calendar date. The first eligible operation per patient in 2020–2025 was selected; laboratory values were assigned using the patient and linked hospitalization and the prespecified windows. Patient identifiers remained in the restricted local linkage layer. Internal checks covered uniqueness of the selected patient, temporal ordering, plausible ranges and the three source-verified duration corrections. These checks are not an external validation of diagnosis or procedure coding. No externally validated code-based case-identification algorithm was used. The frozen extracts, code and source hashes were retained locally; patient-level records are not publicly available under the data-access restrictions described in the main manuscript.

Additional post hoc analyses added during peer review

The original cohort, outcomes, primary model and regression methods were retained. A dated amendment specified the following additions before calculation. Table S3 reports all planned reference-group variants regardless of their estimates. The first excluded six reference-group patients with a documented full-thickness resection outside the rectosigmoid. The second also excluded 15 reference-group patients whose resection multiplicity was missing. Both binary and continuous technique models adjusted for baseline Hb and duration; continuous intervals used HC3 robust standard errors. Overall technique comparisons used 2-df likelihood-ratio or HC3 Wald tests.

Collinearity was described using Spearman correlation of duration with raw organ count and generalized variance inflation factors (GVIFs). GVIFs were calculated from the unweighted design matrix, with the intercept removed from the inverse-cross-product coefficient correlation matrix. Categorical technique and organ-category terms were evaluated as two-degree-of-freedom blocks. We report GVIF, df and GVIF^(1/(2 df)). The joint duration–organ count–technique design is descriptive, not a fitted outcome model. Two additional designs correspond to the actual technique and organ-category analyses. No diagnostic threshold changed the original models.

Postoperative Hb measurement counts were summarized by duration quartiles using type-7 quantiles and right-closed intervals, with ties retained. Counts refer to the same interval from the end of surgery through postoperative day 3. We did not adjust for count because it is measured after the exposure and may reflect postoperative clinical status. Discharge day was calculated as the discharge calendar date of the linked hospitalization minus the operation date, with the operation date defined as day 0. Counts before, on, and after day 3 were reported separately, including by completeness of the Hb pair. Discharge times and interdepartmental transfers were not separately adjudicated.

Numerical calibration summaries used the combined primary model in 416 patients. The original 1000 bootstrap resamples were reproduced with R's Mersenne-Twister generator and seed 20260723. In each resample the model was refitted and its linear predictor was applied to both the resample and the original data. Calibration slope was obtained from logistic regression with an intercept and freely estimated slope for that predictor. Calibration-in-the-large was the intercept from logistic regression with the predictor as an offset (slope fixed at one); positive values indicate underprediction. For each measure, the original apparent value minus the average training-minus-test difference gave the optimism-corrected estimate. Apparent slope 1 and intercept 0 are consequences of in-sample maximum-likelihood fitting, not evidence of predictive validity. Resamples with failed model or calibration convergence were to be excluded jointly and counted; none failed. Decile intervals retained the original Wilson method. Global slope and intercept summaries do not exclude local miscalibration.

AUC contrasts in Table 3 are differences of unrounded apparent AUCs, with percentile intervals from the original 2000 paired bootstrap samples. They are not bootstrap medians. Platelet counts were summarized using available preoperative values; <150 and >400 $\times 10^9$/L are descriptive cut points, not a claimed institutional laboratory interval. For STROBE reporting completeness, unadjusted duration-only logistic and HC3 linear models were subsequently reported on the same 416 complete pairs (Table S3 Panel F). No new primary predictor or outcome was selected during revision.

Post hoc sensitivity to discharge timing and repeated daily measurements

A further dated amendment specified two discharge restrictions and a daily-first-measurement rule before fitting these additional models. Discharge counts and the original event count among early discharges were already known; these analyses were therefore outcome-informed and post hoc. The cohort, primary endpoint and primary model were unchanged. First, patients discharged on or before calendar postoperative day 3 were excluded; the alternative restriction excluded only those discharged before day 3. Discharge was taken from the hospitalization linked to the index operation, without adding later readmissions. Calendar dates do not establish within-day discharge time or sampling opportunity. Conditioning on discharge, which may depend on the operative course and recovery, can introduce selection.

For the measurement sensitivity, eligible Hb samples remained those recorded at or after the derived operation end through calendar postoperative day 3. Within each calendar day the earliest recorded sample time was selected; the nadir was the minimum of these selected values. An exact time tie was to be resolved by the stable source-row order, independently of Hb value; none occurred. All eligible samples had recorded valid times, so no midnight substitution was required. Missing days were not imputed. The unchanged preoperative Hb was used to recalculate decline and the binary threshold. The complete-pair population had to remain identical and the restricted nadir could not be lower than the original nadir. Therefore a non-event could not become an event.

Each sensitivity fitted the original covariates: duration in hours, preoperative Hb, operated-organ count, upper-bowel surgery and bladder surgery. Logistic models used maximum likelihood and model-based standard errors; continuous models used ordinary least squares and HC3 standard errors. Confidence intervals used the estimate plus or minus 1.96 standard errors, with exponentiation for odds ratios. Counts, event frequencies, changed classifications and the number of selected daily measurements by the revised outcome were reported alongside both adjusted duration estimates. Every coefficient and standard error was independently reproduced in Python; source derivation was checked separately. No sampling-count covariate was added because it follows the exposure. These analyses reduce particular opportunities for differential ascertainment but do not remove informative monitoring or establish a clinically validated association.

Software and data handling

Models and resampling were performed in R version 4.6.1 (R Foundation for Statistical Computing, Vienna, Austria). Additional discharge summaries and independent checks of selected regression results were performed in Python 3.12.10. Multiple imputation used mice version 3.19.0. Figures were produced with ggplot2 version 4.0.3, patchwork version 1.3.2, svglite version 2.2.2, ragg version 1.5.2, and scales version 1.4.0. Analyses were performed locally using pseudonymized data.

Table S1. Relationship to previous publications from the same center

Previous publication	Question and reported outcomes	Shared participants among current 424	Interpretation
Mrugała et al., J Clin Med 2026;15:1995 (doi:10.3390/jcm15051995)	Bladder endometriosis surgery within complex pelvic disease; surgical outcomes and descriptive/subgroup perioperative Hb changes	78 (18.4%)	Exact reconstructed participant overlap; 77 shared index operations
Nowak et al., J Clin Med 2026;15:2630 (doi:10.3390/jcm15072630)	Lower colorectal anastomotic dehiscence and rectovaginal fistula; operative risk factors	392 (92.5%)	Exact reconstructed participant overlap; 391 shared index operations
Milnerowicz-Nabzdyk et al., Ginekol Pol 2023;94:698–703 (doi:10.5603/GP.a2022.0122)	Modified segmental bowel-resection technique and bowel leakage after extensive surgery	At least 67 (15.8%)	Confirmed lower bound; full historical membership was unavailable; 67 shared index operations
Union of the three previous cohorts	Any prior cohort membership	At least 402 (94.8%)	Categories overlap; the individual counts must not be added

The current study analyzes an operational Hb decline ≥3 g/dL from baseline to the recorded nadir through postoperative day 3, its association with duration, and exploratory operative-extent contrasts. It is an extension of substantially overlapping institutional series, not an independent validation cohort. Counts refer to participants, not registry rows. The historical 138-patient modified-resection cohort was only partly reconstructable (68 members); therefore 67 shared participants and the 402-person union remain lower bounds. See Section 2.6 and the cited previous publications.

Table S2. Sensitivity analyses

Panel A. Alternative model replacing organ count with uterine and rectosigmoid indicators while retaining upper-bowel and bladder indicators

Predictor	Adjusted OR (95% CI)	p value
Surgery duration, per 60 min	1.61 (1.36 to 1.91)	<0.001
Preoperative Hb, per 1 g/dL	2.28 (1.78 to 2.92)	<0.001
Upper-bowel procedure	1.64 (0.85 to 3.18)	0.141
Bladder procedure	1.03 (0.56 to 1.89)	0.934
Uterine procedure	1.38 (0.86 to 2.22)	0.185
Rectosigmoid procedure	1.77 (0.69 to 4.52)	0.236

Panel B. BMI sensitivity analysis

Panel B1. Logistic model

Predictor	Adjusted OR (95% CI)	p value
Surgery duration, per 60 min	1.64 (1.37 to 1.97)	<0.001
Preoperative Hb, per 1 g/dL	2.65 (2.01 to 3.50)	<0.001
Number of organs involved, per additional organ	1.35 (0.93 to 1.98)	0.119
Upper-bowel procedure	1.23 (0.57 to 2.68)	0.599
Bladder procedure	0.75 (0.37 to 1.53)	0.433
BMI, per 5 kg/m^2	0.55 (0.38 to 0.78)	<0.001

Panel B2. Continuous model

Predictor	Adjusted difference, g/dL (95% CI)	p value
Surgery duration, per 60 min	0.18 (0.12 to 0.24)	<0.001
Preoperative Hb, per 1 g/dL	0.41 (0.33 to 0.50)	<0.001
Number of organs involved, per additional organ	0.05 (−0.10 to 0.20)	0.491
Upper-bowel procedure	0.37 (0.00 to 0.75)	0.049
Bladder procedure	−0.06 (−0.32 to 0.21)	0.667
BMI, per 5 kg/m²	−0.11 (−0.23 to 0.01)	0.069

Panel C. Sensitivity analysis excluding patients with a recorded transfusion

Outcome model	Analysis set	Duration effect (95% CI)	Model summary
Hb decline ≥3 g/dL	n = 374; events = 127	OR 1.60 (1.31 to 1.96)	AUC 0.801
Continuous Hb decline	n = 374	0.17 (0.12 to 0.22) g/dL per 60 min	R^2 0.346

Patients with a recorded transfusion during the index hospitalization were excluded because transfusion can alter the recorded postoperative nadir and therefore the measured decline.

Panel D. Prespecified descriptive summary of conversion to laparotomy

Analysis set	Conversion to laparotomy, n/N (%)
Full cohort	11/424 (2.6%)
Both hemoglobin measurements available	11/416 (2.6%)
One or both hemoglobin measurements unavailable	0/8 (0.0%)

Panel E. Post hoc duration data-quality check (exclusion of surgery durations below 45 minutes)

Exclusion rule	Records removed	Analysis set after exclusion	Duration OR (95% CI)	AUC
Duration <45 min	0 patients with both hemoglobin measurements (0 events)	n = 416; events = 156	1.61 (1.35 to 1.91)	0.770

No accepted surgery duration was below 45 minutes; this refit is therefore identical to the primary analysis.

Panel F. Inverse-probability-weighted analysis addressing missing hemoglobin measurements

Duration effect	Robust covariance	Effective sample size	Maximum absolute standardized difference from the full 424-patient cohort (before and after weighting)
OR 1.61 (1.35 to 1.92)	HC1 sandwich (robust) variance	415.9	0.009 before weighting; 0.000 after weighting

Panel G. Post hoc age and year-of-surgery sensitivity analyses

Added covariate and outcome	Added-covariate estimate (95% CI)	Duration estimate per 60 min (95% CI)
Age per 10 years, Hb decline ≥3 g/dL	OR 1.21 (0.82 to 1.79)	OR 1.62 (1.36 to 1.92)
Age per 10 years, continuous Hb decline	0.12 (−0.03 to 0.27) g/dL	0.20 (0.14 to 0.26) g/dL
Year of surgery, per 1-year increase, Hb decline ≥3 g/dL	OR 1.06 (0.89 to 1.26)	OR 1.67 (1.36 to 2.06)
Year of surgery, per 1-year increase, continuous Hb decline	0.068 g/dL (−0.002 to 0.139)	0.234 (0.158 to 0.310) g/dL

Panel H. Duration-model sensitivity analyses

Sensitivity analysis	Analysis set	Duration or test result	Additional detail
Exclusion of all surgeries involving the bladder	n = 337; events = 123	OR 1.59 (1.31 to 1.94)	AUC 0.750
Addition of a quadratic duration term	n = 416; events = 156	Likelihood-ratio χ^2 = 3.17; df = 1; p = 0.075	Quadratic term, −0.053 (p = 0.058)

In Panel A, the apparent AUC was 0.772. Panel B was restricted to the 395/416 patients (95.0%) with a recorded body mass index (BMI), among whom the primary outcome occurred in 150; BMI was associated with the binary outcome (OR 0.55 per 5 kg/m²), but the continuous coefficient was smaller and imprecise (−0.11 g/dL, p = 0.069), so this exploratory analysis does not establish a protective effect. None of these analyses replaced the primary model. Panel F compares the 416 observed cases, before and after weighting, with the full 424-patient cohort; it is not a comparison of 416 complete cases with the 8 incomplete cases. Confidence intervals from the inverse-probability-weighted (IPW) analysis are conditional on the estimated weights. Hb, hemoglobin; OR, odds ratio; CI, confidence interval; AUC, area under the receiver operating characteristic curve; BMI, body mass index; IPW, inverse-probability weighting.

Table S3. Post hoc analyses added during peer review

The additions were specified in dated post hoc revision amendments before the respective calculations. Early-discharge counts and events were already known before the discharge-sensitivity models were fitted. These analyses are exploratory and do not replace the original analyses.

Panel A. Reference-group sensitivity analyses

Reference definition / contrast	Analysis n / events	Reference n / events	Mean difference in decline, g/dL (95% CI)	OR for decline ≥3 g/dL (95% CI)
Original / discoid	416 / 156	83 / 17	0.33 (0.05 to 0.61)	1.66 (0.73 to 3.74)
Original / segmental	416 / 156	83 / 17	0.40 (0.18 to 0.62)	2.06 (1.06 to 4.00)
Exclude 6 known non-rectosigmoid resections / discoid	410 / 154	77 / 15	0.37 (0.10 to 0.65)	1.76 (0.76 to 4.07)
Exclude 6 known non-rectosigmoid resections / segmental	410 / 154	77 / 15	0.45 (0.22 to 0.67)	2.17 (1.08 to 4.36)
Also exclude 15 reference records with missing multiplicity / discoid	395 / 148	62 / 9	0.37 (0.08 to 0.67)	2.32 (0.89 to 6.06)
Also exclude 15 reference records with missing multiplicity / segmental	395 / 148	62 / 9	0.45 (0.21 to 0.70)	2.92 (1.26 to 6.78)

All models adjust for preoperative Hb and surgery duration. Mean-difference intervals use HC3 robust standard errors; odds-ratio intervals are Wald intervals. In all variants discoid and segmental groups remain 64/20 and 269/119 patients/events. Reference groups are 83/17, 77/15 and 62/9, respectively. Excluding six known resections does not resolve the 15 missing multiplicity records. The second sensitivity may introduce selection related to completeness; neither scenario is preferred on the basis of its result. Overall 2-df tests for each variant are listed below.

Panel A continued. Overall technique contrasts

Variant	Logistic likelihood-ratio p	Continuous HC3 Wald p
Original	0.093	0.0018
Exclude 6	0.080	0.0004
Confirmed reference	0.029	0.0011

Both tests have 2 degrees of freedom; p values are exploratory.

Panel B. Collinearity diagnostics

Design	Term	df	GVIF	GVIF^(1/(2 df))
Joint descriptive design	duration	1	1.386	1.177
Joint descriptive design	organ count	1	1.300	1.140
Joint descriptive design	technique	2	1.227	1.052
Technique model design	duration	1	1.185	1.088
Technique model design	Preoperative Hb	1	1.018	1.009
Technique model design	technique	2	1.199	1.046
Organ-category model design	duration	1	1.255	1.120
Organ-category model design	Preoperative Hb	1	1.021	1.011
Organ-category model design	organ category	2	1.277	1.063

N = 416. GVIF is calculated from the unweighted design matrix, not an IRLS-weighted logistic covariance matrix. The joint design includes duration, raw organ count and the three-level technique factor and is descriptive, not a fitted outcome model. Actual extent model designs include Hb and duration plus either technique or the three-level organ-count category. These are not diagnostics of the full primary model. Spearman correlation between duration and raw organ count was 0.425. No threshold was used to change model specification.

Panel B continued. Overlap between surgical categories

Organs involved	Reference technique	Discoid	Segmental
≤1	45	18	45
2	25	39	140
≥3	13	7	84

Counts describe the 416 patients with both Hb measurements; techniques are mutually exclusive.

Panel C. Postoperative Hb measurement count by duration quartile

Duration, minutes	Patients, n	Measurement count, median (IQR)	Mean count
[60, 170]	112	3 (2–3)	2.63
(170, 220]	104	3 (3–3)	2.81
(220, 286.25]	96	3 (3–3)	2.86
(286.25, 720]	104	3 (3–3)	2.91

Measurement counts use the same window, from the end of surgery through postoperative day 3. Quantiles use R type 7; tied durations remain together and the right endpoint is included. Spearman correlation between duration and count was 0.136. Measurement count was not adjusted for because it follows the exposure and may depend on postoperative clinical status.

Panel D. Discharge relative to the operation

Recorded discharge day	All patients (N = 424)	Complete Hb pair (n = 416)	Incomplete Hb pair (n = 8)
Before POD3	19	19	0
On POD3	37	35	2
After POD3	368	362	6

POD0 is the operation date. All 424 hospitalization records had discharge dates. Median time from operation to discharge was 5 calendar days (IQR 4–6); median preoperative stay was 2 days (IQR 2–3). Among 56 patients discharged through POD3, a nadir and complete pair were available in 54; 5/54 (9.3%) met the endpoint. Discharge times and interdepartmental transfers were not separately adjudicated. These descriptive data cannot distinguish truncated sampling from clinical selection for early discharge.

Panel E. Additional descriptive findings

Finding	Result
Preoperative Hb <12 g/dL	41/419 (9.8%)
Preoperative Hb <13 g/dL	152/419 (36.3%)
Transfusion among incomplete Hb pairs	0/8
Endpoint with missing resection multiplicity	17/37 (45.9%)
Endpoint with available resection multiplicity	139/379 (36.7%)
Missing multiplicity: low composite burden, endpoint events / patients	5/13 (38.5%)
Missing multiplicity: intermediate composite burden, endpoint events / patients	8/17 (47.1%)
Missing multiplicity: high composite burden, endpoint events / patients	4/7 (57.1%)
Preoperative platelets, median (IQR), $\times 10^9$/L	265 (232–314.5); n = 419
Preoperative platelets, mean ± SD; range, $\times 10^9$/L	277.5 ± 71.3; 95–628
Platelets <150 $\times 10^9$/L	8/419 (1.9%)
Platelets >400 $\times 10^9$/L	22/419 (5.3%)

The composite burden cross-tabulation is restricted to the 37 patients with both Hb measurements but missing multiplicity; high burden in this subset can arise only from involvement of at least three organs. It is descriptive, with no subgroup model. Hb thresholds are strict inequalities and share the same measurement window. Platelet cut points are descriptive conventions, not a verified institutional reference interval. The platelet distribution and low thrombocytopenia frequency do not establish the explanation for a neutral adjusted estimate.

Panel F. Unadjusted duration associations added for STROBE reporting

Outcome	Patients, n	Estimate per hour (95% CI)
Hb decline ≥3 g/dL	416	OR 1.53 (1.32 to 1.76)
Continuous Hb decline	416	0.21 g/dL (0.14 to 0.28)

Each model contains only an intercept and duration. Logistic intervals are Wald intervals; continuous intervals use HC3 robust standard errors. Added post hoc for reporting completeness; the primary adjusted estimates remain in Table 3. Independent Python reproduction agreed with the R estimates and standard errors.

Panel G. Post hoc sensitivity to discharge timing and daily measurement selection

Analysis	Patients, n	Events, n (%)	Duration OR per hour (95% CI)	Continuous decline per hour, g/dL (95% CI)
Original primary model (reference)	416	156 (37.5%)	1.61 (1.35 to 1.91)	0.20 (0.13 to 0.26)
Exclude discharge on or before POD3	362	151 (41.7%)	1.54 (1.28 to 1.84)	0.17 (0.10 to 0.23)
Exclude discharge before POD3	397	154 (38.8%)	1.59 (1.33 to 1.90)	0.18 (0.12 to 0.25)
First recorded Hb per calendar day	416	153 (36.8%)	1.61 (1.35 to 1.91)	0.20 (0.14 to 0.26)

All models use the original covariates (duration, preoperative Hb, organ count, upper-bowel and bladder surgery); continuous intervals use HC3 standard errors. POD0 is the operation calendar date. The restrictions change only the sensitivity population or daily sample selection, not the primary analysis. The first-daily rule changed classification in 3/416 patients: 3 from event to non-event and 0 in the opposite direction. The mean number of selected measurements was 2.79 in the revised event group and 2.58 in the revised non-event group. Excluding early discharges can introduce selection; first-daily sampling does not equalize the number of observed days. All results are reported regardless of direction.

Table S4. Apparent decile calibration and post hoc bootstrap calibration summaries

Predicted-risk decile	Patients, n	Events, n	Mean predicted, %	Observed, % (Wilson 95% CI)
1	42	5	6.6	11.9 (5.2–25.0)
2	42	8	13.4	19.0 (10.0–33.3)
3	42	8	19.7	19.0 (10.0–33.3)
4	42	7	25.5	16.7 (8.3–30.6)
5	42	7	31.1	16.7 (8.3–30.6)
6	42	15	37.6	35.7 (23.0–50.8)
7	41	28	44.8	68.3 (53.0–80.4)
8	41	18	52.9	43.9 (29.9–59.0)
9	41	25	65.2	61.0 (45.7–74.3)
10	41	35	80.2	85.4 (71.6–93.1)

Deciles refer to the unchanged primary combined model in 416 patients (156 events). They are in-sample summaries; local departures remain despite near-zero average calibration-in-the-large. The bootstrap optimism-corrected slope was 0.949 and calibration-in-the-large was +0.003 (intercept in logistic regression with predicted logit as an offset and slope fixed at one). Positive values indicate underprediction. All 1000 resamples succeeded. Apparent slope 1 and intercept 0 follow from maximum-likelihood fitting in the same sample and are not evidence of external calibration. See Supplementary Methods for the resampling definition. These analyses do not propose a clinical prediction tool.

Table S5. Component-level missingness, multiple imputation, and missing-not-at-random analyses

Panel A. Availability of the two hemoglobin components

Pattern	n/N (%)
Both preoperative Hb and postoperative nadir observed	416/424 (98.1%)
Preoperative Hb only	3/424 (0.7%)
Postoperative nadir only	5/424 (1.2%)
Neither component observed	0/424 (0.0%)

Panel B. Complete-case, weighting, and multiple-imputation estimates

Analysis	Primary-outcome frequency	Duration OR per 60 min (95% CI)	Continuous duration effect per 60 min (95% CI)
Complete-case analysis	156/416 (37.5%); 95% CI, 32.8–42.3%	1.61 (1.35 to 1.91)	0.20 (0.13 to 0.26) g/dL
Inverse-probability weighting of patients with both hemoglobin measurements	—	1.61 (1.35 to 1.92)	—
Multiple imputation under a missing-at-random assumption	37.6% (95% CI, 33.0% to 42.3%)	1.58 (1.33 to 1.88)	0.19 (0.13 to 0.25) g/dL

Multiple imputation used m = 50 datasets, 20 iterations, predictive mean matching, and 5 donors. Preoperative Hb and postoperative nadir were imputed as separate components; decline and binary outcomes were derived afterward. The fraction of missing information was 0.010 for the duration log-odds coefficient and 0.003 for the continuous duration coefficient. MAR, missing at random; OR, odds ratio; CI, confidence interval; Hb, hemoglobin.

Panel C. Pattern-mixture delta scenarios

Δ applied to imputed preoperative Hb, g/dL	Δ applied to imputed postoperative nadir, g/dL	Outcome frequency	Duration OR (95% CI)	Continuous effect, g/dL per 60 min (95% CI)
−0.5	+0.0	37.4%	1.59 (1.33 to 1.88)	0.19 (0.13 to 0.25)
−0.5	−0.5	37.6%	1.58 (1.33 to 1.88)	0.19 (0.13 to 0.25)
−0.5	−1.0	37.7%	1.58 (1.33 to 1.88)	0.19 (0.13 to 0.25)
−0.5	−1.5	37.8%	1.58 (1.33 to 1.88)	0.19 (0.13 to 0.25)
+0.0	+0.0	37.6%	1.58 (1.33 to 1.88)	0.19 (0.13 to 0.25)
+0.0	−0.5	37.8%	1.58 (1.33 to 1.88)	0.19 (0.13 to 0.25)
+0.0	−1.0	37.9%	1.58 (1.33 to 1.87)	0.19 (0.13 to 0.25)
+0.0	−1.5	38.1%	1.58 (1.33 to 1.87)	0.19 (0.13 to 0.25)
+0.5	+0.0	37.8%	1.59 (1.34 to 1.88)	0.19 (0.13 to 0.25)
+0.5	−0.5	38.0%	1.58 (1.33 to 1.88)	0.19 (0.13 to 0.25)
+0.5	−1.0	38.1%	1.58 (1.33 to 1.88)	0.19 (0.13 to 0.25)
+0.5	−1.5	38.2%	1.58 (1.33 to 1.88)	0.19 (0.13 to 0.25)

Deltas were applied only to imputed values. A negative postoperative-nadir delta assumes a lower unobserved nadir and therefore a larger decline. Across the 12 scenarios, outcome frequency ranged from 37.4% to 38.2%, duration ORs from 1.58 to 1.59, and all continuous duration estimates rounded to 0.19 g/dL per 60 minutes; no 95% interval included the null. With only eight incomplete pairs, comparing patients with and without complete hemoglobin data cannot reliably identify the missingness mechanism. MNAR, missing not at random.

Table S6. Complete coefficient sets for the binary and continuous duration models

Panel A. Logistic model for Hb decline ≥3 g/dL

Predictor	Adjusted OR (95% CI)	p value
Surgery duration, per 60 min	1.61 (1.35 to 1.91)	<0.001
Preoperative Hb, per 1 g/dL	2.32 (1.81 to 2.98)	<0.001
Number of organs involved, per additional organ	1.25 (0.87 to 1.80)	0.218
Upper-bowel procedure	1.30 (0.61 to 2.77)	0.499
Bladder procedure	0.77 (0.39 to 1.54)	0.461

Panel B. Continuous Hb decline model

Predictor	Adjusted mean difference, g/dL (95% CI)	p value
Surgery duration, per 60 min	0.20 (0.13 to 0.26)	<0.001
Preoperative Hb, per 1 g/dL	0.40 (0.32 to 0.49)	<0.001
Number of organs involved, per additional organ	0.07 (−0.07 to 0.22)	0.313
Upper-bowel procedure	0.34 (−0.03 to 0.70)	0.072
Bladder procedure	−0.08 (−0.34 to 0.18)	0.544

Panel A included 416 patients and 156 events. Panel B included 416 patients and used HC3 robust standard errors. The preoperative-Hb coefficient is mathematically coupled to the change outcome and is not interpreted as an independent biological effect; Table 3 reports the analyses that exclude preoperative Hb and the bootstrap AUC comparisons. Hb, hemoglobin; OR, odds ratio; CI, confidence interval; AUC, area under the receiver operating characteristic curve.

Table S7. Surgical-group profiles and additional model information

Panel A. Unadjusted group profiles

Exposure variable	n	Hb decline ≥3 g/dL, n/N (%)	Hb decline, median (IQR), g/dL	Duration, median (IQR), min
Rectosigmoid technique	—	—	—	—
No recorded discoid/segmental rectosigmoid resection, including shaving-only procedures	83	17/83 (20.5%)	2.10 (1.60–2.80)	175 (135–238)
Discoid resection	64	20/64 (31.2%)	2.45 (2.10–3.10)	168 (140–200)
Segmental resection	269	119/269 (44.2%)	2.80 (2.20–3.40)	250 (195–320)
Number of full-thickness bowel resections	—	—	—	—
No resection	62	9/62 (14.5%)	2.10 (1.53–2.60)	172 (131–220)
One resection	266	102/266 (38.3%)	2.70 (2.10–3.30)	210 (170–269)
Multiple resections	51	28/51 (54.9%)	3.10 (2.30–3.95)	295 (232–342)
Number of organs involved	—	—	—	—
≤1 organ	108	28/108 (25.9%)	2.40 (1.87–3.02)	180 (145–240)
Two organs	204	76/204 (37.3%)	2.55 (2.00–3.23)	208 (165–260)
≥3 organs	104	52/104 (50.0%)	2.95 (2.20–3.70)	292 (230–361)

Panel B. Information added beyond preoperative Hb and surgery duration

Panel B1. Continuous Hb decline

Exposure variable	n	p value for the exposure variable	Δ R^2
Rectosigmoid technique	416	0.002	0.020
Number of full-thickness bowel resections	379	<0.001	0.030
Number of organs involved	416	0.066	0.010
Anastomotic height	240	0.476	0.001

Panel B2. Binary Hb decline endpoint

Exposure variable	n	p value for the exposure variable	Δ AUC	Δ Brier score
Rectosigmoid technique	416	0.093	0.008	−0.0024
Number of full-thickness bowel resections	379	0.034	0.014	−0.0040
Number of organs involved	416	0.186	0.003	−0.0021
Anastomotic height	240	0.673	0.002	−0.0001

Panel C. Continuous models without adjustment for surgery duration

Contrast	Patients, n	Adjusted difference in Hb decline, g/dL (95% CI)
Rectosigmoid technique (reference: no recorded discoid/segmental rectosigmoid resection, including shaving-only procedures)	—	—
Discoid resection	416	0.29 (−0.01 to 0.59)
Segmental resection	416	0.64 (0.40 to 0.87)
Number of full-thickness bowel resections (reference: no resection)	—	—
One resection	379	0.55 (0.29 to 0.80)
Multiple resections	379	1.10 (0.70 to 1.50)
Number of organs involved (reference: ≤1 organ)	—	—
Two organs	416	0.22 (−0.003 to 0.44)
≥3 organs	416	0.70 (0.42 to 0.97)
Anastomotic height above the anal verge	—	—
Per 5-cm increase	240	−0.10 (−0.36 to 0.16)

Panel A contains unadjusted summaries. In Panel B, each exploratory model — that is, an exposure variable added to preoperative Hb and surgery duration — is compared with the model containing preoperative Hb and surgery duration alone, fitted in the same patients. A positive Δ AUC and a negative Δ Brier score both indicate better performance. Panel C is adjusted for preoperative Hb but not for surgery duration. Missing resection multiplicity was not imputed, and no anastomotic-height cut point was examined. Rectosigmoid technique denotes the rectosigmoid technique group defined in the Methods. Hb, hemoglobin; IQR, interquartile range; CI, confidence interval; AUC, area under the receiver operating characteristic curve.

Table S8. Post hoc operative-burden and preoperative hemostatic-marker analyses

Panel A. Ordered operative-burden trend

Outcome and adjustment	Estimate per category increase (odds ratio or g/dL; 95% CI)	p value
Hb decline ≥3 g/dL, unadjusted	OR 1.93 (1.36 to 2.74)	<0.001
Hb decline ≥3 g/dL, adjusted for preoperative Hb	OR 2.38 (1.61 to 3.50)	<0.001
Hb decline ≥3 g/dL, adjusted for preoperative Hb and surgery duration	OR 1.48 (0.96 to 2.28)	0.079
Continuous decline, unadjusted	+0.46 g/dL (0.27 to 0.64)	<0.001
Continuous decline, adjusted for preoperative Hb	+0.51 g/dL (0.34 to 0.68)	<0.001
Continuous decline, adjusted for preoperative Hb and surgery duration	+0.30 g/dL (0.13 to 0.48)	<0.001

Operative burden was coded 0 = low, 1 = intermediate, and 2 = high; the definitions are given in Table 5.

Panel B. Preoperative platelet count, APTT, and INR

Added marker and scale	Patients, n	Adjusted OR for Hb decline ≥3 g/dL (95% CI)	Δ AUC	Adjusted difference in continuous Hb decline, g/dL (95% CI)
Platelet count, per 50 × 10^9/L	416	1.01 (0.86 to 1.18)	−0.0002	+0.028 (−0.034 to 0.089)
APTT, per 5 s	415	0.94 (0.64 to 1.40)	+0.0006	−0.011 (−0.161 to 0.139)
INR, per 0.1	413	1.13 (0.84 to 1.52)	+0.0024	−0.030 (−0.143 to 0.083)

Each marker was added separately to the same reference model, in the subset of patients for whom that marker was available. The combined three-marker block had a likelihood-ratio p value of 0.822 and changed the apparent AUC by +0.00237. Bootstrap 95% CIs for the combined block were −0.0011 to 0.0166 for Δ AUC and −0.0055 to −0.0001 for Δ Brier score; both changes were negligible in magnitude. APTT and INR had narrow observed ranges, so these analyses do not address clinically important coagulopathy. Hb, hemoglobin; OR, odds ratio; CI, confidence interval; AUC, area under the receiver operating characteristic curve; APTT, activated partial thromboplastin time; INR, international normalized ratio.
